# Long-term dietary replacement of fishmeal and fish oil in diets for rainbow trout (*Oncorhynchus mykiss*): Effects on growth, whole body fatty acids and intestinal and hepatic gene expression

**DOI:** 10.1371/journal.pone.0190730

**Published:** 2018-01-24

**Authors:** Viviana Lazzarotto, Françoise Médale, Laurence Larroquet, Geneviève Corraze

**Affiliations:** INRA - UMR 1419 “Nutrition Métabolisme Aquaculture”, Aquapôle, Saint Pée-sur-Nivelle, France; Universitat Politècnica de València, SPAIN

## Abstract

The effects of replacing fishmeal and fish oil with a plant-based diet were studied in juvenile (10g) and ongrowing (250-350g) rainbow trout from first-feeding. Feed-related differences in the intestinal and hepatic transcriptome were examined in juveniles after 7 months of feeding at 7°C. Based on microarray results obtained for juveniles, the expression of selected genes related to lipid, cholesterol and energy metabolisms, was assessed by RT-qPCR in ongrowing trout after 6 additional months of feeding at 17°C. Plasma glucose and cholesterol, lipid content and fatty acid profile of whole body were analyzed at both stages. After 7 months at 7°C, all juveniles reached the same body weight (10g), while at 13 months ongrowing fish fed the totally plant-based diet exhibited lower body weight (234 *vs* 330-337g). Body lipid content was higher in juveniles fed the totally plant-based diet (13.2 *vs* 9.4–9.9%), and plasma cholesterol was about 2-times lower in trout fed the plant-based diets at both stages. Fatty acid profile mirrored that of the respective diet, with low proportions of long-chain n-3 polyunsaturated fatty acids in fish fed plant-based diets. Genes involved in protein catabolism, carbohydrate metabolism and trafficking were down-regulated in the intestines of juveniles fed the plant-based diets. This was not true for ongrowing fish. Genes involved in lipid and cholesterol metabolisms were up-regulated in the livers of fish fed plant-based diets for both stages. In this study, feeding trout a totally plant-based diet from first-feeding affect a relatively low proportion of metabolism-related genes. In the longer term, when fish were reared at a higher temperature, only some of these changes were maintained (*i*.*e*. up-regulation of lipid/cholesterol metabolism). Although the plant-based diets tested in this study had no major deficiencies, small adjustments in the feed-formula are needed to further optimize growth performance while sparing marine resources.

## Introduction

Aquaculture production has increased almost 12-fold [1] over the last three decades and now provides consumers with a consistent supply of high-quality seafood. This rapid growth in production has resulted in an increased demand for aqua-feeds. Availability of traditional aqua-feed ingredients, including fish meal (FM) and fish oil (FO), has not increased with demand, and today readily available alternative sources of proteins and lipids are required [2]. Many studies have evaluated the effects of replacing FM and FO with plant ingredients, and as a result, the FM and FO contents of commercial aqua-feeds have decreased in recent years [3]. However, extensive use of plant products has several known disadvantages, particularly with respect to differences in amino acid (AA), cholesterol and fatty acid (FA) compositions between plant and marine feed ingredients, as well as the presence of anti-nutritional factors found in plant feedstuffs [4]. These differences in composition can interfere with feed utilization, and certain negative effects are observed with high levels of substitution. For example, growth performances were reduced in juvenile Atlantic salmon (*Salmo salar*) fed a diet in which 80% FM and 70% FO were replaced by plant ingredients [5]. Decreased growth was also observed in juvenile rainbow trout (*Oncorhynchus mykiss*) fed a completely plant-based diet, compared to trout that were fed a diet containing marine ingredients [6]. The authors of these studies suggested that the lower growth observed was mainly related to the substitution of FM, rather than the replacement of FO.

The replacement of marine ingredients, and in particular the substitution of FO with plant ingredients, is known to drastically modify FA composition of the diet. While none of the vegetable oils contain n-3 long chain polyunsaturated fatty acids (n-3 LC-PUFAs) such as eicosapentaenoic acid (EPA, 20:5 n-3) and docosahexaenoic acid (DHA, 22:6 n-3), they are rich in other FAs, mainly 18:0, 18:1, 18:2 n-6 and 18:3 n-3 [7]. The FA composition of fish tissue changes in response to the FA composition of the diet, as shown in previous studies in which dietary FM and FO were replaced with plant ingredients [5,8,9].

In addition to traditional measures of the effects of substitution of marine ingredients with plant sources, (*i*.*e*. growth performance and tissue FA composition), recent advances in functional genomics (*i*.*e*. gene expression) have provided new opportunities to better understand the basic molecular pathways involved in the response of fish to new diets [8,10,11]. Determining the patterns of gene expression through the study of tissue transcriptomes (mRNA expression) provides extensive information about how dietary ingredients are perceived by fish. This analysis provides a molecular snapshot of the physiological response of specific tissues [6,10,12]. The effects of replacing different proportions of FM and/or FO with plant ingredients on gene expression in fish tissues has been widely studied [5,6,8,13,14,15]. However, among the studies that focused on the liver, very few investigated the transcriptional effects of total and simultaneous replacement of marine ingredients with plant sources. Panserat et al. [6] presented evidence that the replacement of both FM and FO with plant ingredients in the diet of rainbow trout juveniles induced changes in the hepatic expression of genes involved in nucleic acid and glucose metabolisms, as well as in the expression of genes involved in lipid and protein metabolisms. In another study, carried out on European sea bass (*Dicentrarchus labrax*) there was stimulation of the lipogenic pathways in the livers of fish fed a totally plant-based diet [15].

While less studied than the liver, a growing number of reports have examined intestinal gene expression in fish in response to different levels of dietary replacement of marine ingredients with plant products [13,16,17,18,19]. A study on the effects of dietary FO replacement by vegetable oils on the intestinal transcriptome of Atlantic salmon, revealed that lipid and energy metabolisms were the functional categories most affected by diet [16]. Conversely, in a study where Atlantic cod (*Gadus morhua*) juveniles were fed diets that replaced FO with increasing proportions of vegetable oils (33% up to 100%), no major diet-induced metabolic changes were detected in the intestine [18]. In the same study, genes potentially able to alter cellular proliferation and death or change the structural property of intestinal muscle, were found to be up-regulated in the intestines of cod fed a diet with vegetable oils [18]. Replacing 30% of FM with plant ingredients in the diet of Atlantic halibut (*Hippoglossus hippoglossus*) induced an up-regulation in the intestinal expression of genes involved in immune responses and in xenobiotic detoxification, and down-regulation of genes involved in lipid transport, protein synthesis and cell growth [20]. Substituting 50% FM with plant protein in Atlantic salmon diet, resulted in changes in the intestinal expression of genes involved in protein and energy metabolism, as well as in genes involved in cell proliferation and apoptosis [13].

To date, the limited number of studies that have investigated the impact of total and concomitant substitution of FM and FO with plant products on the metabolic response of fish tissue have focused their scope to a relatively short- or middle-term experiment [6,15].

The primary objectives of this study were to investigate the diet-induced changes in the intestinal and hepatic transcriptome of juvenile rainbow trout after 7 months of feeding plant-based diets from first-feeding and to establish whether these changes would be maintained when trout are reared at optimum temperature (17°C) over a longer period (6 additional months).

## Materials and methods

### Feeding trial and experimental diets

The experiment was carried out in strict accordance with EU legal frameworks relating to the protection of animals used for scientific purposes (Directive 2010/63/EU) and according to the National Guidelines for Animal Care of the French Ministry of Research (Decree N° 2001–464, May 29, 2001). It was approved by the Ethics Committee of INRA (INRA 2002–36, April 14, 2002) and the scientist in charge of the experiment received training and personal authorization (N°B64 10 003).

The experimental plan of the present study can be divided into two sequential periods:

1^st^ period: from 1^st^ feeding to 7-months and 2^nd^ period: from 7 to 13 months. For clarity, throughout this manuscript, all 7-month old trout will be defined as “juveniles”, in accordance with the definition given by Kendall et al. [21] and 13-month old trout will be defined as “ongrowing fish”.

#### First period

The first period of the feeding trial took place at the INRA fish facilities of Lees-Athas (Permit N° A64 104 1). At the beginning of the experiment, rainbow trout (*O*. *mykiss*) fry with mean weight of 135 ± 1 mg, were randomly distributed among 12 tanks (310 fish per tank). Throughout the trial, 50-L tanks were used and water flow was set to ensure an oxygen concentration above 90% saturation. Fish were exposed to natural photoperiod condition and the water temperature was 7 ± 1°C. This low water temperature is ideal for trout during the first part of their development because it limits the risk of diseases, as is often done in commercial fish farms [22]. Fish were kept under these rearing conditions for 7 months. Throughout the trial, dead fish (if any) were removed daily and weighed. Rates of fish survival were assessed as a percentage of the initial number of fish which survived. The fish in each tank were bulk-weighed every 3 weeks in order to check the evolution of body weight as the experiment progressed.

During this first period of 7 months from the time of first feeding, trout were fed either a marine M-diet (based on FM and FO), or a commercial-like C-diet (46% of FM and 69% of FO replaced by plant ingredients), or a totally plant-based V-diet (100% plant proteins and vegetable oils) with four tanks of fish provided with each diet. These three experimental diets, presented in Tables 1 and 2, were the same as those previously described in Lazzarotto et al. [23] (pellets size: 1–3 mm). Fish were fed by hand, four times per day, until apparent satiation.

**Table 1:** Ingredients and composition of the experimental diets.

	*1*^*st*^ *period*			*2*^*nd*^ *period*		
*Diets*	M	C	V	M	C	V
*Ingredients (%)*						
**Fish meal** *	**65.2**	**30.0**	**0.0**	**54.3**	**30.0**	**0.0**
Corn gluten	0.0	13.2	24.0	0.0	10.2	18.0
Soybean meal 48	0.0	6.1	2.0	0.0	6.3	4.3
Wheat gluten	0.0	10.0	22.0	0.0	5.0	12.1
Soy protein concentrate	0.0	10.2	20.0	0.0	3.5	18.1
White lupin	0.0	0.4	2.5	0.0	6.5	5.0
Peas	0.0	4.1	0.0	0.0	6.9	2.4
Rapseed meal 00	0.0	6.2	2.3	0.0	6.3	9.8
Extruded whole wheat	21.1	1.3	0.0	30.1	7.2	2.8
**Fish oil** **	**11.7**	**8.1**	**0.0**	**13.6**	**8.0**	**0.0**
Rapeseed oil	0.0	8.1	6.7	0.0	8.0	7.3
Linseed oil	0.0	0.0	6.7	0.0	0.0	7.3
Palm oil	0.0	0.0	3.6	0.0	0.0	3.0
Min.-Vit. premix ***	2.0	2.0	2.0	2.0	2.0	2.0
Soy lecithin	0.0	0.0	2.0	0.0	0.0	2.0
L-lysine	0.0	0.3	1.5	0.0	0.1	1.5
L-methionine	0.0	0.01	0.3	0.0	0.0	0.3
CaHPO_4_.2H_2_0 (18% P)	0.0	0.0	2.9	0.0	0.0	2.6
Attractant mix	0.0	0.0	1.5	0.0	0.0	1.5
*Composition (% DM)*						
Dry matter (DM, %)	94.3	95.3	95.5	93.8	95.2	95.0
Crude protein	48.9	53.3	52.9	44.4	46.3	47.2
Crude fat	21.5	22.1	21.8	22.0	24.2	24.5
Starch	20.5	11.5	8.2	20.0	11.5	8.0
Energy (kJ/g DM)	23.0	24.2	24.1	23.9	24.3	25.1
Total sterols	0.70	0.55	0.36	0.68	0.51	0.41

*Origin co-fishery products—all species

** Origin co-fishery products—sardines

*** Min.-Vit. premix: Mineral premix (g or mg kg^−1^ diet): calcium carbonate (40% Ca), 2.15 g; magnesium oxide (60% Mg), 1.24 g; ferric citrate, 0.2 g; potassium iodide (75% I), 0.4 mg; zinc sulphate (36% Zn), 0.4 g; copper sulphate (25% Cu), 0.3 g; manganese sulphate (33% Mn), 0.3 g; dibasic calcium phosphate (20% Ca, 18% P), 5 g; cobalt sulphate, 2 mg; sodium selenite (30% Se), 3 mg; KCl, 0.9 g; NaCl, 0.4 g (UPAE, INRA).

Vitamin premix (IU or mg kg^−1^ diet): DL-a tocopherol acetate, 60 IU; sodium menadione bisulphate, 5 mg; retinyl acetate, 15,000 IU; DL-cholecalciferol, 3000 IU; thiamin, 15 mg; riboflavin, 30 mg; pyridoxine, 15 mg; B12, 0.05 mg; nicotinic acid, 175 mg; folic acid, 500 mg; inositol, 1000 mg; biotin, 2.5 mg; calcium pantothenate, 50 mg; choline chloride, 2000 mg (UPAE, INRA).

M: marine FM-FO-based diet

C: commercial-like FM-FO & plant-based diet

V: experimental 100% plant-based diet

**Table 2:** Proportions of the main fatty acids (% of total FA) in experimental diets.

	*1*^*st*^ *period*			*2*^*nd*^ *period*		
	M	C	V	M	C	V
*Fatty acid*						
**SAT**	30.8	20.9	18.5	26.6	18.2	17.6
**MUFA**	33.2	41.9	38.3	28.9	42.9	37.9
18:2 n-6	3.2	12.5	21.5	2.9	12.0	21.4
20:4 n-6	0.7	0.4	0.0	0.8	0.4	0.0
**PUFA n-6**	4.3	13.1	21.5	4.3	12.8	21.4
18:3 n-3	1.1	4.8	21.3	0.8	4.5	22.7
18:4 n-3	2.1	1.2	0.0	2.3	1.3	0.0
20:5 n-3	11.1	6.7	0.0	14.7	8.3	0.0
22:5 n-3	1.1	0.7	0.0	1.7	1.1	0.0
22:6 n-3	6.7	4.2	0.0	9.9	5.5	0.0
**PUFA n-3**	23.3	18.1	21.3	30.8	21.4	22.7

M: marine FM-FO-based diet

C: commercial-like FM-FO & plant-based diet

V: experimental 100% plant-based diet

SAT: saturated fatty acids

MUFA: monounsaturated fatty acid

PUFA: polyunsaturated fatty acid

#### Second period

For the second period of the trial, fish were transferred to the INRA experimental facilities in Donzacq (Permit N° A40 2281). On arrival, fish within each dietary group (*e*.*g*. M-, C- or V-fed trout) were split into 3 tanks with 150 trout per tank (9 tanks in total). The fish were acclimated to the new rearing conditions for two weeks and fed with their respective diets. At the start of the second experimental period, the average weight of fish was 12.5 g. Throughout the trial, 200-L tanks were used (maximum stocking densities: 26 kg/m^3^) and the oxygen saturation was greater than 90%. Fish were exposed to natural photoperiod condition and the water temperature was 17 ± 1°C. This water temperature corresponds to the thermal preferendum for growth of rainbow trout [22]. Fish were maintained under these rearing conditions for 6 months. As it was done during the first period of the trial, dead fish (if any) were removed daily and weighed. Rates of fish survival were assessed as a percentage of the initial number of fish that survived. The fish in each tank were bulk-weighed every 3 weeks in order to check the evolution of body weight as the experiment progressed.

Diets used throughout this second period of the trial contained the same ingredients as those used for the first period. In order to adapt the formulation to different stages and fish size, the proportions of these ingredients were slightly different among diets used in the first and the second part of the trial. Details about the ingredients and composition of the experimental diets are given in Table 1 and the proportions of the main FA in the diets in Table 2. The pellet size was adapted to fish size and during the second part, ongrowing fish received 4–5 mm diets. Fish were hand-fed twice a day to apparent visual satiety, in order to avoid any uneaten pellets, and feed distributed was recorded. Feed efficiency (FE) was calculated as follows: FE = g weight gain/g dry feed given.

### Sampling

#### First period

By the end of the 7-month feeding period, 12 fish were taken from each tank 48 hours after the last meal, euthanized by immersion in a 6% benzocaine solution (anesthetic overdose), and weighed. Fish were then separated into two pools of 6 fish each, and stored at -20°C for whole body lipid content and whole body FA profile analyses.

An additional 4 fish were collected from each tank. These fish were then anaesthetized and their blood was drawn from the caudal vein and collected in heparinized syringes and centrifuged (3 000 *g*, 5 min). The plasma recovered was immediately frozen and kept at -20°C until further analysis. Fish were then euthanized by anesthetic overdose, dissected and the intestine and liver were sampled for gene expression analysis (*e*.*g*. transcriptomic and RT-qPCR). Samples were immediately frozen in liquid nitrogen and stored at -80°C until analysis.

#### Second period

By the end of the experiment, 5 fish per tank were sampled 48 hours after the last meal, euthanized by immersion in a 6% benzocaine solution, pooled and stored at -20°C for whole body lipid content and whole body FA profile analyses.

For an additional 5 fish per tank, blood was removed from the caudal vein and plasma was recovered as previously described. Fish were then euthanized by anesthetic overdose, dissected and the intestine and liver were sampled for gene expression analysis (RT-qPCR). Samples were immediately frozen in liquid nitrogen and stored at -80°C until analysis.

### Chemical analysis of the diets

Feeds were ground before determination of proximate composition according to standard procedures [24]. The chemical composition of the diets was analyzed as follows: dry matter (DM) after drying at 105°C for 24 h, lipid content by petroleum ether extraction (Soxtherm, Gerhardt, Konigswinter, Germany), protein content (N× 6.25) by the Kjeldahl method after acid digestion, gross energy in an adiabatic bomb calorimeter (IKA, Heitersheim Gribheimer, Germany), and starch by enzymatic method [25].

Total sterols in diets were measured according to the Liebermann-Burchard method [26].

### Plasma metabolite analysis

Plasma glucose (Glucose RTU, BioMérieux, Marcy-l’Etoile, France) and cholesterol (CHOL100, Sobioda) levels were determined using commercial kits adapted to a micro-plate format, according to the recommendations of the manufacturer.

### Lipid and fatty acid analysis

Whole body fish from each tank were pooled and ground. Total lipids were extracted after homogenization in dichloromethane/methanol (2:1, v/v), with 0.01% butylated hydroxytoluene (BHT) as an antioxidant, using an Ultra-Turrax (IKA-Werke, Germany) tissue disrupter and quantified gravimetrically [27]. Fatty acid methyl esters (FAME) were prepared from diet and fish lipid extracts according to Shantha & Ackman [28]. FAMEs were then analyzed in a Varian 3900 gas chromatograph equipped with a fused silica DB Wax capillary column (30m x 0.25 mm internal diameter, film thickness 0.25 μm; JW Alltech, France). Injection volume was 1 μl, using helium as carrier gas (1 ml/min). The temperatures of the injector and the flame ionization detector were 260°C and 250°C, respectively. The thermal gradient was as follows: 100–180°C at 8°C/min, 180–220°C at 4°C/min and a constant temperature of 220°C for 20 min. Fatty acids were identified with reference to a known standard mixture (Sigma, St Louis, MO, USA) and peaks were integrated using Varian Star Chromatography Software (Star Software, version 5). Individual FAs were expressed as a percentage of total FAME identified.

Amounts of EPA+DHA (g fish^-1^) were calculated taking into account, for each tank, mean body lipid content (g fish^-1^) and the percentages of EPA and DHA in body lipids. Values were expressed as mean per dietary treatment.

### RNA isolation

Among all of the collected tissue, intestine (mid-gut) and liver samples were chosen by selecting the fish with a body weight closest to the mean body weight per tank. Total RNA was extracted from individual intestines (mid gut) and from the livers of juveniles (n = 2 per tank, n = 8 per dietary treatment) and ongrowing fish (n = 2 per tank, n = 6 per dietary treatment).

Prior to extraction, samples were homogenized using Precellys24 (Bertin Technologies, Montigny-le-Bretonneux, France) in 2 ml tubes containing TRIzol^®^ reagent (Invitrogen, Carlsbad, CA) and 2.8 millimeter ceramic beads, 2 × 10 s, separated by 15 s off, at 5,000 rpm. The extraction of total RNA was then performed according to the manufacturer’s recommendations. The quantities of extracted RNA were analyzed using a spectrophotometer (ND-1000, Nanodrop). The quality of extracted RNA was assessed on the basis of RNA Integrity Number (RIN) using a Bioanalyzer (Agilent Technologies, Kista, Sweden).

### Microarray hybridization and analysis

The microarray analysis was performed on juveniles at the end of the first period of the trial. Samples of RNA from individual intestines (mid gut) and from the livers of juveniles (8 individual samples per diet), were analyzed using a custom-commercial 8X60K oligoarray (Agilent Technologies, Massy, France; Gene Expression Omnibus (GEO) Accession No. GPL15840).

Cy3-labelled experimental cRNA samples were generated using the Agilent “One-Color Microarray-based Gene Expression Analysis” (Low Input Quick Amp Labeling-LIQA) kit, as previously described in detail [29]. Cy3-labelled cRNA sample yield (>0.825μg cRNA) and specific activity (>6pmol of Cy3/μg of cRNA) were verified using a NanoDrop ND-1000. Forty-eight samples (two tissues x three dietary treatments x eight replicates) were processed. For each reaction, 600ng of Cy3-cRNA were fragmented and hybridized on a sub-array, following the LIQA kit (Agilent) instructions. The hybridization reactions were allowed to run for 17h in a rotating hybridization oven (65°C) prior to washing according to the manufacturer’s instructions. Samples were randomized, preventing those from the same dietary treatment from being overrepresented in a particular batch in order to avoid unintentional biases. Slides were scanned with an Agilent Scanner (Agilent DNA Microarray Scanner, Agilent technologies, Massy, France) using the standard parameters for an 8x60K gene expression oligoarray (3μm– 20 bits). Data were then obtained with the Agilent Feature Extraction software (10.7.1.1) according to an appropriate gene expression (GE) protocol (GE1_107_Sep09). The data set was deposited in NCBI’s GEO (GSE84985).

### Real Time q-PCR (RT-qPCR)

For each experimental condition, six samples of intestine (mid gut) and liver from individual juveniles and ongrowing fish were used as biological replicates for RT-qPCR analysis. In juveniles the six samples were chosen based on the best RIN obtained (RIN ≥9.0).

In addition to validating differentially expressed genes obtained from the microarray analysis, we also analyzed the expression of additional candidate genes related to lipid metabolism in the livers (*Elovl5* and *HMGCR*) and intestines (*Δ-6desaturase*, *Elovl2* and *Elovl5*) of juveniles, because they were found to be affected by dietary replacement of FO by vegetable oils in many other studies [6,8,15,29,30].

The same genes were also studied in the intestines (mid gut) and livers of ongrowing fish (six samples per tissue per experimental condition). Primer design was performed using Primer 3 software. Specific primer pairs were designed with an overlapping intron when possible, using known trout sequences in nucleotide databases (GeneBank and INRA-Sigenae). Database Accession Numbers and the sequences of forward and reverse primers used for each gene are provided in Table 3.

**Table 3:** Primer sequences of genes selected for analysis by RT-q PCR.

Gene	*Primer 5’-3’ (FW)*	*Primer 5’-3’ (RV)*	*Annealing temperature*, *°C*
CTSZ	GGAGCCCTTCATCAACCACA	TTGTTGGTCCACTGCCTGTT	60
CTSS	TTTGCCTCATTGCGTGTTCC	GTCTTTCATCAGCTGGCCCT	60
FAAH	TCCCTGTCTCCACGGTAACA	AACAGCCTCTCCACCTCTCT	60
CYP51A1	CCCGTTGTCAGCTTTACCA	GCATTGAGATCTTCGTTCTTGC	60
HMGCR	GAACGGTGAATGTGCTGTGT	GACCATTTGGGAGCTTGTGT	60
DHCR7	GTAACCCACCAGACCCAAGA	CCTCTCCTATGCAGCCAAAC	60
MDH2	TTGACATTGCCCACACACCT	AGATCATCACGGGTCATGCC	60
COX5B	AGATCACTGCCACGACACTATG	CTTTCCTTTCTTCAGTGCCTGC	60
COX7A2L	CCCTTGATGTGGACTGGCAA	GAGGCTTCACACCGAGTACA	60
Elovl2	TGTGGTTTCCCCGTTGGATGCC	ACAGAGTGGCCATTTGGGCG	59
Elovl5	GAACAGCTTCATCCATGTCC	TGACTGCACATATCGTCTGG	59
Δ6-desaturase	AGGGTGCCTCTGCTAACTGG	TGGTGTTGGTGATGGTAGGG	59
***Reference genes***			
EF1α	TCCTCTTGGTCGTTTCGCTG	ACCCGAGGGACATCCTGTG	59
β-actin	GATGGGCCAGAAAGACAGCTA	TCGTCCCAGTTGGTGACGAT	59

CTSZ, cathepsin Z; CTSS, cathepsin S; FAAH, fatty acid amide hydrolase; CYP51A1, cytochrome P450, family 51, subfamily A, polypeptide 1; HMGCR, 3-hydroxy-3-methylglutaryl-CoA reductase; DHCR7, 7-dehydrocholesterol reductase; MDH2, malate dehydrogenase 2, NAD (mitochondrial); COX5B, cytochrome c oxidase, subunit Vb; COX7A2L, cytochrome c oxidase, subunit VIIa polypeptide 2 like; Elovl2, polyunsaturated fatty acid elongase 2; Elovl5, polyunsaturated fatty acid elongase 5; Δ6-desaturase, delta-6-desaturase; EF1α, eukaryotic translation initiation factor 1 alpha 1; β-actin, beta actin.

For the RT-qPCR, total RNA (1μg) was reverse-transcribed to cDNA with the SuperScript III RNase H reverse transcriptase kit (Invitrogen, Carlsbad, CA, USA) using oligo dT Primers. Real-time PCR was performed in the iCycler iQ TM (BIO-RAD, Hercules, CA, USA). Quantitative PCR analyses for gene expression were performed on 10μl of the RT reaction mixture using the iQ TM SYBR ^®^ Green Supermix (BIO-RAD, Hercules, CA, USA). The total volume of the PCR reaction was 25μl containing 200nM of each primer. Thermal cycling was initiated with incubation at 95°C (90s) for hot-start iTaq TM DNA polymerase activation.

Thirty-five steps of PCR were performed, each consisting of a heating step at 95°C (20s) for denaturing, and at 59°C 30s for annealing and extension. Melting curves were systematically monitored following the final PCR cycle (with a gradient of 0.5°C/10s from 55°C to 94°C) to ensure that only one fragment was amplified. Samples without reverse transcriptase and samples without RNA were run for each reaction as negative controls. Expression of two reference genes, *e*.*g*. elongation factor-1α (*EF1-α*) and beta actin (*β-actin*), was quantified for both tissue types in samples from juveniles and ongrowing fish. mRNA levels for all target genes studied in the liver were initially normalized with the housekeeping gene *EF1-α*, previously used as a reference gene in salmonids [31], and the expression levels were calculated according to threshold cycle (ΔΔCt). However, because none of the two reference genes tested for the intestine were stable for all experimental groups, mRNA levels of target genes studied were normalized following the method proposed by Matz et al.[32]. Moreover, to validate this analytical choice, we also tested the data of liver gene expression and compared them with the data obtained through the “classic” method (housekeeping-gene normalization). Since the results obtained with these two approaches were the same, we concluded that our chosen methodology was appropriate. In order to compare gene expression levels in the two types of tissue studied, we normalized data on mRNA levels for all target genes studied in both the intestine and the liver following the method proposed by Matz et al. [32]. The results were analyzed using the MCMC.qpcr R-package that implements a generalized linear mixed model analysis of RT-qPCR data, based on the lognormal-Poisson model.

### Statistical analysis and data mining

Tanks were used as the experimental unit for data on growth, body lipid content and fatty acid profile. Individual fish were the experimental unit for data on plasma parameters and gene expression, since no tank-related effect was observed during the experiment. Data for biometric parameters, lipid content and fatty acids are presented as mean ± standard deviation (SD). Data were analyzed statistically using the R software (version 2.14.0) and the Rcmdr package. The normality and the homogeneity of variance of the variables were tested with Shapiro-Wilk’s and Levene’s tests, respectively. When both conditions were satisfied, a one-way ANOVA (*p-value* <0.05) was performed to assess the effects of the diets. The variables with non-parametric distribution (some fatty acids) were normalized with an arcsin transformation. If the criteria (normality and homogeneity) were still not met, a non-parametric test was used for the analysis.

Data from microarray analyses were normalized and analyzed statistically using GeneSpring software (12.6, Agilent). Data were scale-normalized using the median value of each array to identify genes differentially expressed between conditions. Differentially expressed genes were obtained by one-way ANOVA (diet, *p-value* <0.05). For all genes found to be differentially expressed, GO ontologies were obtained using the Expression Analysis Systematic Explorer (EASE) software, version 2.0 [33]. Significant GO enrichment was tested using EASE software, with Benjamini-Hochberg correction (score <0.05). Data from RT-qPCR were analyzed by one-way ANOVA (diet, *p-value* <0.05) followed by a Tukey’s post hoc test (*p-value* <0.05).

## Results

### Survival and growth

**Fig 1:**
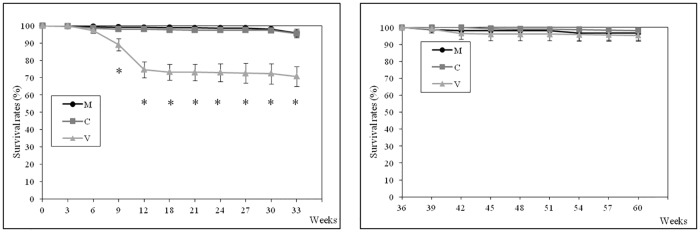
Survival during the first (on the left) and the second period (on the right) of the feeding trial. Data are expressed as mean ± standard deviation. Fish survival is expressed as % of survivors in relation to the initial number of fish in each tank, at each experimental period. *, statistically significant differences between V- *vs* C- and M-fed fish (one-way ANOVA, *p<0*.*05*).

**Fig 2:**
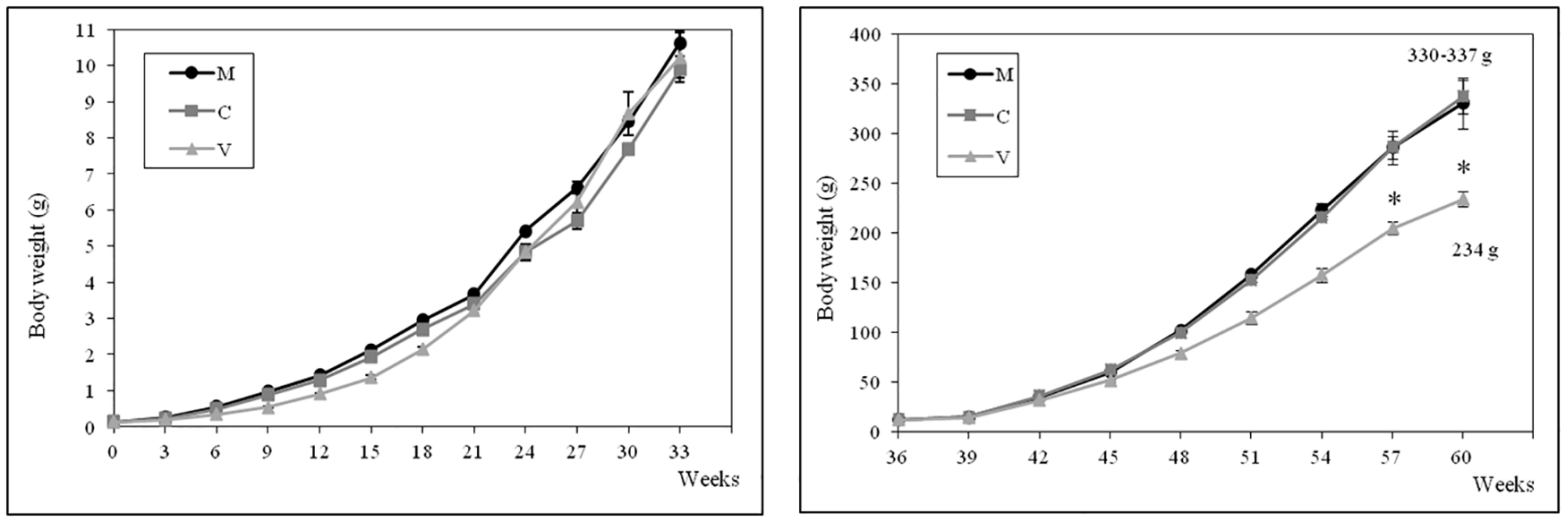
Body weight of rainbow trout during the first (on the left) and the second period (on the right) of the feeding trial. Data are expressed as mean ± standard deviation. Fish were bulk weighed every 3 weeks during the whole duration of the feeding trial. *, statistically significant differences between V- vs C- and M-fed fish (one-way ANOVA, *p<0*.*05*).

Survival and body weight data for juveniles and ongrowing fish are presented in Figs 1 and 2, respectively. At the end of the first period of the trial (7 months after the first feeding time), significantly lower survival rates were observed in the V-fed group compared to groups fed the C- and M-diets (65% *vs* 95%), mainly due to the high mortality recorded for the V-fed group during the first twelve weeks. During the second period of the trial, no significant differences were found in survival rates (96–98% throughout the period) irrespective of the dietary treatment.

Despite the slower growth recorded for the V-fed fish during the first twelve weeks (Fig 2), at the end of the first period of the trial (7 months) mean body weight was not significantly different between the three groups (10 ± 1g).

At the end of the second period of the trial (13 months) significantly lower body weights were recorded for fish fed the V-diet (234 ± 7g) compared to the M-fed (330 ± 25g) and the C-fed (337 ± 17g) groups. No significant differences in body weight were found between the C- and M-fed groups (Fig 2). Lower but not statistically different (*p* = 0.11) values of feed efficiency (FE = g weight gain/g dry feed given) were observed with the V-diet (1.04 ± 0.06) when compared to the M-(1.19 ± 0.13) and C-fed fish (1.22 ± 0.07).

### Plasma metabolites

**Fig 3:**
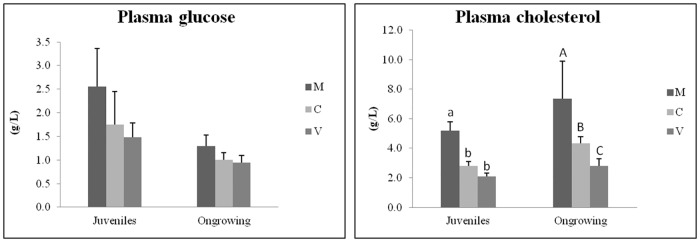
Plasma metabolites in juveniles and ongrowing fish (g/L). Data are means ± standard deviation (n = 16 individuals /dietary treatment for juveniles; n = 15 individuals/dietary treatment for ongrowing fish). Differences between diets were analyzed by one-way ANOVA, followed by Tukey’s test. Values that do not share a common letter are significantly different (*p<0*.*05*), with lower and upper case letters indicating differences between dietary groups within juveniles and ongrowing fish, respectively.

Plasma metabolites were measured 48h after feeding in juveniles (7-month feeding trial) and ongrowing fish (13-month feeding trial). Plasma glucose levels were not significantly different between dietary treatments, in juveniles or in ongrowing fish (Fig 3). Significantly lower plasma cholesterol levels were found in both juveniles and ongrowing fish fed the plant-based diets when compared to the M-fed group (Fig 3).

### Whole body lipids and FA profile

At the end of the first period of the trial, significantly higher lipid contents (Table 4) were found in the whole body of juveniles fed the V-diet (+23%), compared to those fed the M- or the C-diets. No significant differences were recorded in ongrowing fish (15–16% body lipids) (Table 4).

**Table 4:** Total lipid whole body content (% of fresh weight) and proportions (% of total FA) of the main fatty acids in juveniles and ongrowing fish.

*Diets*	M		C		V		
	Mean	SD	Mean	SD	Mean	SD	*p-value*
*Juveniles*							
**Lipids** (%)	9.40^b^	0.38	9.94^b^	1.52	13.20^a^	0.88	*<0*.*01*
**SAT**	24.7^a^	0.4	18.2^b^	0.5	16.5^c^	1.1	*<0*.*001*
**MUFA**	34.1^c^	0.3	42.8^a^	0.1	39.1^b^	0.6	*<0*.*001*
18:2 n-6	3.5^c^	0.2	12.6^b^	0.2	18.7^a^	0.4	*<0*.*001*
20:4 n-6	0.8	0.02	0.5	0.04	0.5	0.05	*<0*.*001*
**PUFA n-6**	4.9^c^	0.2	14.3^b^	0.2	22.4^a^	0.5	*<0*.*001*
18:3 n-3	1.0^c^	0	3.8^b^	0.1	11.3^a^	0.3	*<0*.*001*
18:4 n-3	1.6^b^	0.1	1.1^b^	0.0	5.5^a^	0.5	*<0*.*001*
20:5 n-3	8.3^a^	0.2	4.2^b^	0.1	1.0^c^	0.1	*<0*.*001*
22:5 n-3	2.1^a^	0.8	1.1^b^	0.1	0.2^c^	0.0	*<0*.*001*
22:6 n-3	14.5^a^	0.4	9.4^b^	0.2	2.2^c^	0.2	*<0*.*001*
**PUFA n-3**	29.1^a^	0.8	20.7^b^	0.2	21.1^b^	1.1	*<0*.*001*
*Ongrowing*							
**Lipids** (%)	15.1	1.1	16.2	0.8	15.2	0.9	*ns*
**SAT**	30.7^a^	0.5	22.5^b^	0.5	18.0^c^	0.1	*<0*.*001*
**MUFA**	31.3^c^	0.7	43.1^a^	0.3	38.2^b^	0.3	*<0*.*001*
18:2 n-6	3.0^c^	0.1	11.8^b^	0.3	19.3^a^	0.2	*<0*.*001*
20:4 n-6	0.7^a^	0.0	0.4^c^	0.0	0.5^b^	0.0	*<0*.*001*
**PUFA n-6**	4.5^c^	0.1	13.2^b^	0.3	20.8^a^	0.2	*<0*.*001*
18:3 n-3	0.8^c^	0.0	3.5^b^	0.1	13.2^a^	0.2	*<0*.*001*
18:4 n-3	1.5^b^	0.0	0.8^c^	0.0	4.0^a^	0.0	*<0*.*001*
20:5 n-3	8.9^a^	0.4	4.0^b^	0.1	0.8^c^	0.0	*<0*.*001*
22:5 n-3	2.5^a^	0.2	1.3^b^	0.0	0.2^c^	0.0	*<0*.*001*
22:6 n-3	10.4^a^	0.6	6.5^b^	0.2	1.9^c^	0.3	*<0*.*001*
**PUFA n-3**	25.8^a^	1.2	17.2^c^	0.3	20.8^b^	0.3	*<0*.*001*

M, marine FM-FO-based diet; C, commercial-like FM-FO & plant-based diet; V, experimental 100% plant-based diet; SAT, saturated fatty acid; MUFA, monounsaturated fatty acid; PUFA, polyunsaturated fatty acid; ns, not significant. Statistical differences were determined by one-way ANOVA followed by Tukey’s HSD comparison test. Mean values that do not share a common letter are significantly different (*p<0*.*05*). Juveniles: n = 4; ongrowing fish: n = 3.

The whole body fatty acid composition of juveniles and ongrowing fish (Table 4) reflected that of the respective diets (Table 2). Lower percentages of saturated fatty acids (SAT) were found in fish fed the plant-based diets (C and V), compared to M-fed fish. Levels of monounsaturated fatty acids (MUFA) were higher in fish fed the C- and V-diets compared to those fed the M-diets. Trout fed the V-diets exhibited the highest levels of n-6 PUFA, mainly due to the high percentages of 18:2 n-6. The arachidonic acid (ARA, 20:4 n-6) levels were higher in fish fed the M-diet, compared to those fed the C- and the V-diets. 18:3 n-3 levels were higher in fish fed diets containing plant ingredients (V>C>M), whereas lower n-3 LC-PUFA percentages (1% EPA and 2% DHA) were found in fish fed the V-diets than in fish fed the C-or M-diets.

Amounts of EPA and DHA expressed per g fish^-1^ (Table 5) were lower in body lipids when plant ingredients were included in the diet (M>C>V). Amounts of EPA + DHA were higher in ongrowing fish than in juveniles, irrespective of the dietary treatment.

**Table 5:** Amounts of EPA + DHA (g fish^-1^) at different developmental stages in response to the experimental diet.

	EPA + DHA			
	*Juveniles*		*Ongrowing fish*	
*Diets*	Mean	SD	Mean	SD
Diet-M	0.20^a^	0.01	8.9^A^	1.12
Diet-C	0.08^b^	0.004	5.4^B^	0.37
Diet-V	0.01^c^	0.002	0.9^C^	0.07

EPA, eicosapentaenoic acid; DHA, docosahexaenoic acid; M, marine FM-FO-based diet; C, commercial-like FM-FO & plant-based diet; V, experimental 100% plant-based diet. Statistical differences were determined by one-way ANOVA followed by Tukey’s HSD comparison test. Mean values that do not share a common letter are significantly different (*p<0*.*001*), with lower and upper case letters indicating differences between dietary groups within juveniles and ongrowing fish, respectively. Juveniles: n = 4; ongrowing fish: n = 3.

### Microarray analysis in juveniles

#### Intestinal transcriptome

**Fig 4:**
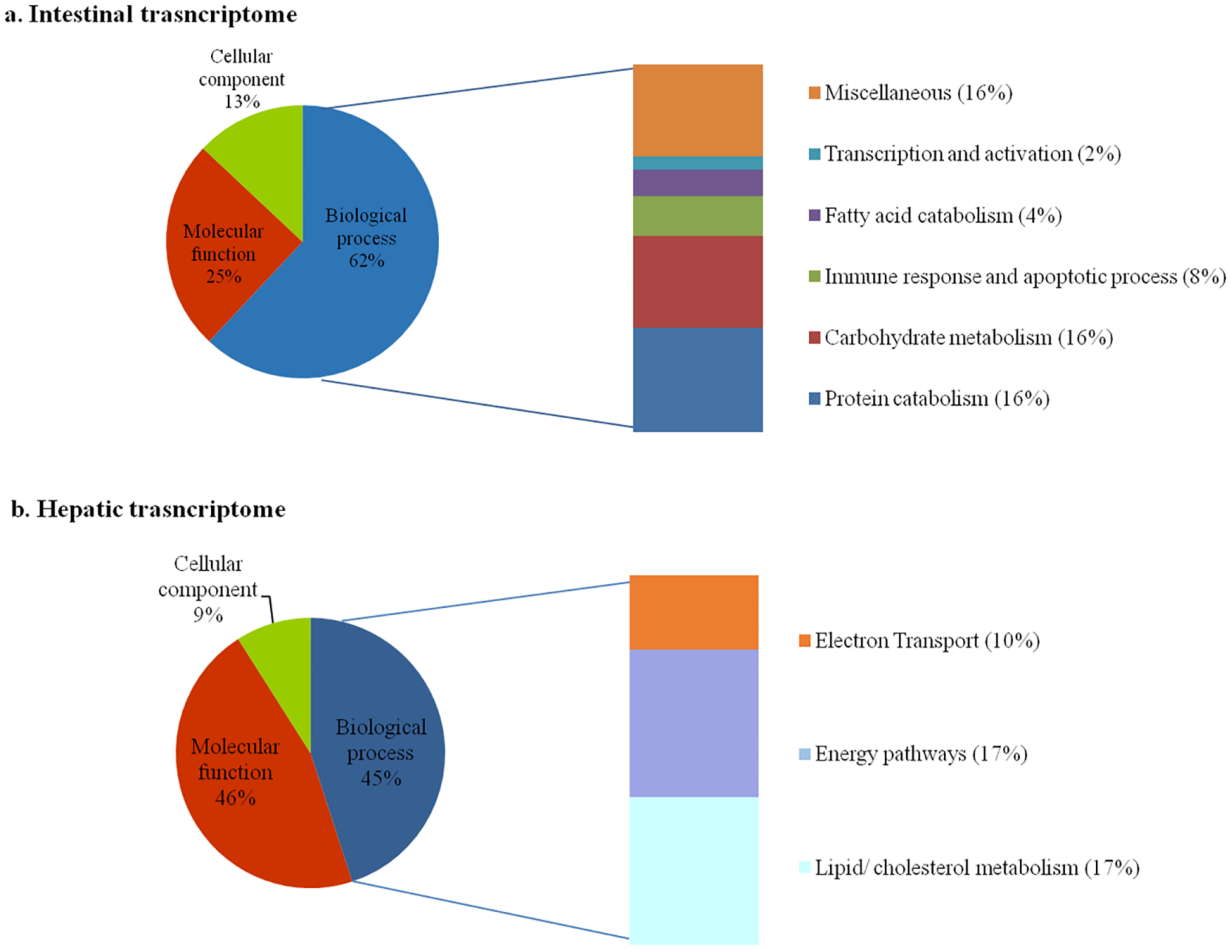
a-b. Intestinal (a) and hepatic (b) transcriptome: proportions of different GO-categories represented by differentially expressed genes obtained by a one-way ANOVA (factor: *diet*, FDR 0.05).

Analysis by one-way ANOVA of the intestinal transcriptome of juvenile rainbow trout showed that 143 genes were significantly differentially expressed in response to the dietary treatments. Of these, 45 had an assigned gene annotation (S1 Table). The GO enrichment analysis (EASE score <0.05) highlighted changes in expression of genes involved in biological processes (62%), molecular function (25%) and cellular component (13%) as shown in Fig 4a.

Concerning biological processes (Table 6, Fig 4a), seven genes involved in protein degradation (16% of annotated genes) were found to be down-regulated in the intestine of fish fed the plant-based diets, with a more pronounced down-regulation in the C-fed group. The same down-regulation trend was observed for seven genes involved in carbohydrate metabolism (16% of annotated genes). The GO enrichment also indicated down-regulation of four genes involved in the immune response and apoptotic process (8% of annotated genes), as well as two genes involved in fatty acid catabolism (4% of annotated genes) in fish fed the plant-based diets, compared to those fed the M-diet. One gene involved in transcription and activation processes (2% of annotated genes) was up-regulated in fish fed the C-diet, compared to fish fed the other two experimental diets.

**Table 6:** Impact of dietary treatments on the intestinal transcriptome of juveniles related to the expression of genes involved in GO biological processes.

			Fold Change (FC)			*Significance* *p-value*
Probe Name	Gene Symbol	Description	*C vs M*	*V vs M*	*V vs C*	
*Protein catabolism*						
*TC99247*	CTSH	cathepsin H	- 5.1	- 2.6	+ 2.0	*0*.*035*
*CUST_68_PI425708691*	CTSL2	cathepsin L2	- 11.1	- 4.0	+ 2.8	*0*.*041*
***CUST_24029_PI425536763***	**CTSZ**	**cathepsin Z**	**- 5.7**	**- 2.9**	**+ 2.0**	***0*.*013***
*TC106655*	DPP7	dipeptidyl-peptidase 7	- 2.2	- 2.1	+ 1.0	*0*.*036*
*CUST_20321_PI425536763*	FOLH1	folate hydrolase	- 3.9	- 1.8	+ 2.2	*0*.*041*
*TC110997*	LGMN	Legumain	- 9.0	- 4.5	+ 2.0	*0*.*041*
*CUST_25677_PI425536763*	ENPEP	glutamyl aminopeptidase (aminopeptidase A)	- 13.8	- 1.9	+ 7.2	*0*.*041*
*Carbohydrate metabolism*						
*CUST_21158_PI425536763*	MAN2B1	mannosidase, alpha, class 2B, member 1	- 7.1	- 5.4	+ 1.3	*0*.*012*
*CUST_17398_PI425536763*	FUCA1	fucosidase, alpha-L- 1, tissue	- 4.4	- 2.8	+ 1.5	*0*.*035*
*CUST_12758_PI425536763*	FUCA2	fucosidase, alpha-L- 2, plasma	- 8.3	- 3.8	+ 2.2	*0*.*025*
*TC114862*	GLB1	galactosidase, beta 1	- 11.4	- 3.5	+ 3.3	*0*.*016*
*TC104967*	NAGA	N-acetyl galactosaminidase, alpha	- 11.2	- 3.8	+ 3.0	*0*.*042*
*TC108468*	NEU1	neuraminidase 1	- 25.8	- 7.2	+ 3.6	*0*.*025*
**TC123951**	**PFKFB3**	**6-phosphofructo-2-kinase/fructose-2,6-biphosphatase 3**	**- 2.8**	**- 3.0**	**+ 1.1**	***0*.*042***
*Immune response/apoptotic process*						
***CUST_8157_PI425536763***	**CTSS**	**cathepsin S**	**- 11.0**	**- 3.8**	**+ 2.9**	***0*.*041***
*CUST_14242_PI425536763*	MPO	Myeloperoxidase	-1.5	- 4.9	- 3.3	*0*.*035*
*CUST_7188_PI425536763*	BAD	BCL2-associated agonist of cell death	- 2.3	- 1.3	+ 1.8	*0*.*045*
*TC104795*	MAP3K7	mitogen-activated protein kinase kinase kinase 7	- 1.1	- 1.5	+ 1.6	*0*.*049*
*Fatty acid catabolism*						
*TC94736*	CPT1A	carnitine palmitoyltransferase 1A (liver)	+1.4	+1.0	-1.4	*0*.*045*
***TC121737***	**FAAH**	**fatty acid amide hydrolase**	**-18.6**	**-3.0**	**+6.2**	***0*.*041***
*Transcription and activation*						
*TC125816*	PQBP1	polyglutamine binding protein 1	+1.3	-1.1	-1.4	*0*.*042*
*Miscellaneous*						
*CUST_17716_PI425536763*	MXD4	max dimerization protein 4	-1.7	-1.6	+1.1	*0*.*025*
*CUST_1243_PI425536763*	POLR2F	polymerase (RNA) II (DNA directed) polypeptide F	1.4	-1.1	-1.5	*0*.*039*
*TC118891*	PRPSAP2	phosphoribosyl pyrophosphate synthetase-associated protein 2	+1.4	-1.0	-1.4	*0*.*025*
*CUST_9923_PI425536763*	TTC4	tetratricopeptide repeat domain 4	+1.8	+1.5	-1.2	*0*.*039*
*CUST_6882_PI425536763*	RENBP	renin binding protein	-4.4	-2.5	+1.8	*0*.*036*
*TC95545*	ASAH1	N-acylsphingosine amidohydrolase 1	-2.6	-1.9	+1.4	*0*.*041*
*CUST_15445_PI425536763*	ASH2L	ash2 (absent, small, or homeotic)-like	-2.2	-1.7	+1.3	*0*.*049*

Genes tested by RT-q PCR are in bold.

GO analysis of genes from the molecular function and cellular component categories showed an overall down-regulation in response to the C-diet when compared to M- and V-fed fish (S1 Table).

#### Hepatic transcriptome

The one-way ANOVA revealed that 53 genes were differentially expressed in the liver in response to the dietary treatments. Of these, only 22 had an assigned gene annotation (S2 Table). The GO enrichment analysis (EASE score <0.05) highlighted changes in expression of genes involved in biological processes (45%, Fig 4b and Table 7). Of these, the pathways most affected by the dietary treatments were lipid/cholesterol metabolism (4 genes, 18% of annotated genes), energy pathways (4 genes, 18% of annotated genes) and electron transport (2 genes, 9% of annotated genes). Among the genes related to lipid metabolism, we observed up-regulation of those involved in LC-PUFA bioconversion and cholesterol biosynthesis with the V-diet compared to the other treatment groups. Genes involved in energy pathways were also up-regulated in fish fed the V-diet as well as the two genes involved in electron transport. Differential regulation in response to the diet was observed for genes belonging to the GO molecular function category (46% of enriched genes, Fig 4b) with, notably, global up-regulation of genes involved in macromolecule biosynthesis with the V-diet compared to the other two experimental groups (V>M>C; S2 Table). Diet also affected the expression of two genes belonging to the GO cellular components category (9% of enriched genes, Fig 4b and S2 Table).

**Table 7:** Impact of dietary treatments on the hepatic transcriptome of juveniles related to the expression of genes involved in GO biological processes.

			Fold Change (FC)			*Significance* *p-value*
Probe name	Gene Symbol	Description	*C vs M*	*V vs M*	*V vs C*	
*Lipids/Cholesterol Metabolism*						
**CUST_14393_PI425536763**	**Elovl2**	**polyunsaturated fatty acid elongase**	**+ 1.4**	**+ 2.6**	**+ 1.8**	***0*.*049***
**TC130473**	**CYP51A1**	**cytochrome P450, family 51, subfamily A, polypeptide 1**	**+ 2.3**	**+ 4.1**	**+ 1.8**	***0*.*046***
**TC130143**	**DHCR7**	**7-dehydrocholesterol reductase**	**+ 2.3**	**+ 3.8**	**+ 1.6**	***0*.*049***
CUST_9914_PI425536763	TM7SF2	transmembrane 7 superfamily member 2	+ 2.6	+ 5.7	+ 1.8	*0*.*030*
*Energy pathways*						
CUST_21841_PI425536763	ATP5B	ATP synthase, H+ transporting mitochondrial F1 complex, beta subunit	- 1.0	+ 1.5	+ 1.5	*0*.*030*
CUST_20841_PI425536763	ATP5C1	ATP synthase, H+ transporting, mitochondrial F1 complex, gamma polypeptide 1	+1.1	+ 1.4	+ 1.3	*0*.*040*
**CUST_11055_PI425536763**	**MDH2**	**malate dehydrogenase 2, NAD (mitochondrial)**	**+1.0**	**+ 1.7**	**+ 1.7**	***0*.*030***
TC114386	UQCRC1	ubiquinol-cytochrome c reductase core protein I	-1.0	+ 1.7	+ 1.7	*0*.*030*
*Electron Transport*						
**TC105004**	**COX5B**	**cytochrome c oxidase subunit Vb**	**-1.2**	**+1.4**	**+ 1.6**	***0*.*038***
**TC99046**	**COX7A2L**	**cytochrome c oxidase subunit VIIa polypeptide 2 like**	**-1.2**	**+1.5**	**+ 1.9**	***0*.*027***

Genes tested by RT-q PCR are in bold.

### RT-qPCR

**Fig 5:**
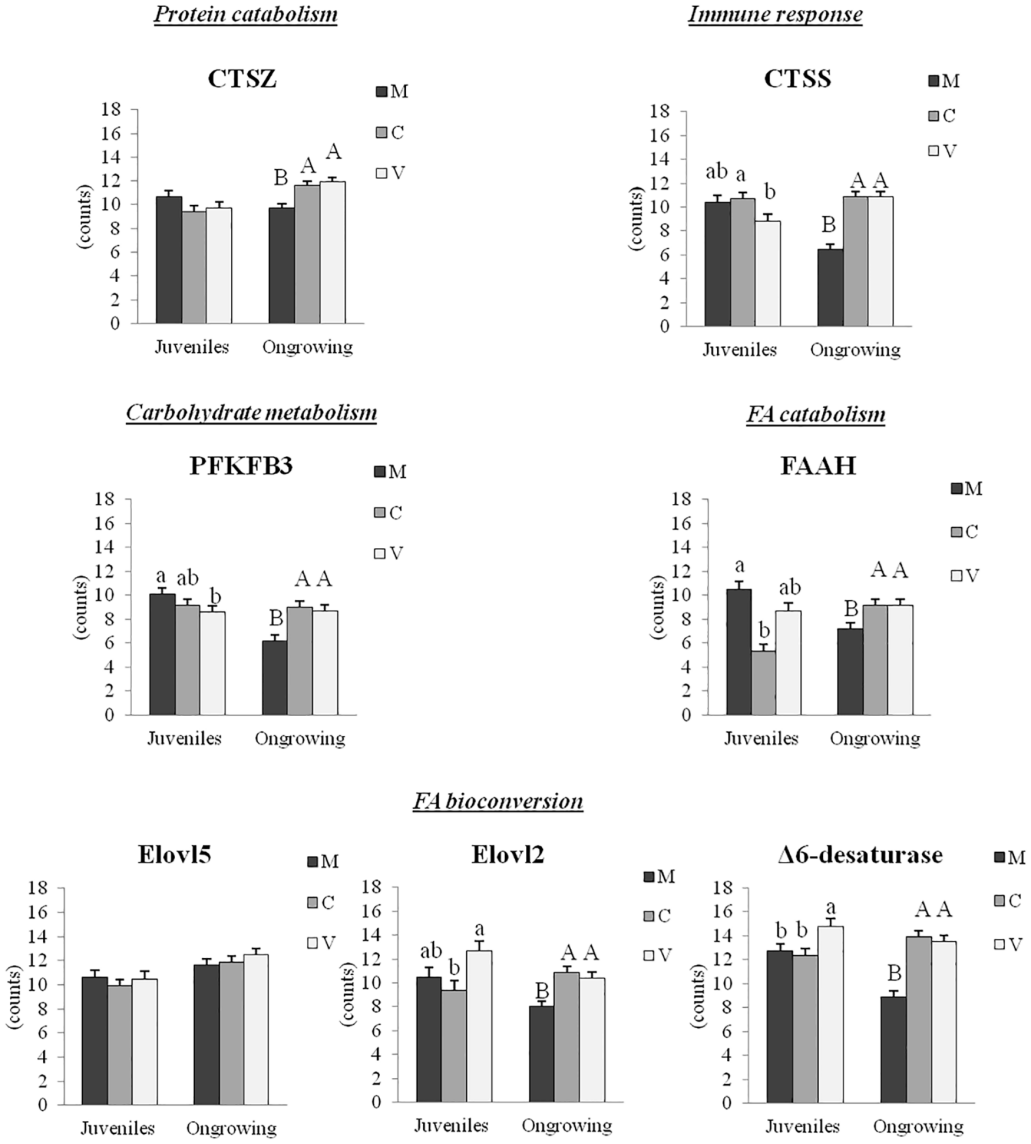
Intestinal gene expression in juveniles and ongrowing fish (RT-qPCR). Data are mean ± S.D. (n = 6 individuals/treatment). Differences between diets were analyzed by one-way ANOVA, followed by Tukey’s test. Values that do not share a common letter are significantly different (*p<0*.*05*), with lower and upper case letters indicating differences between dietary groups within juveniles and ongrowing fish, respectively.

**Fig 6:**
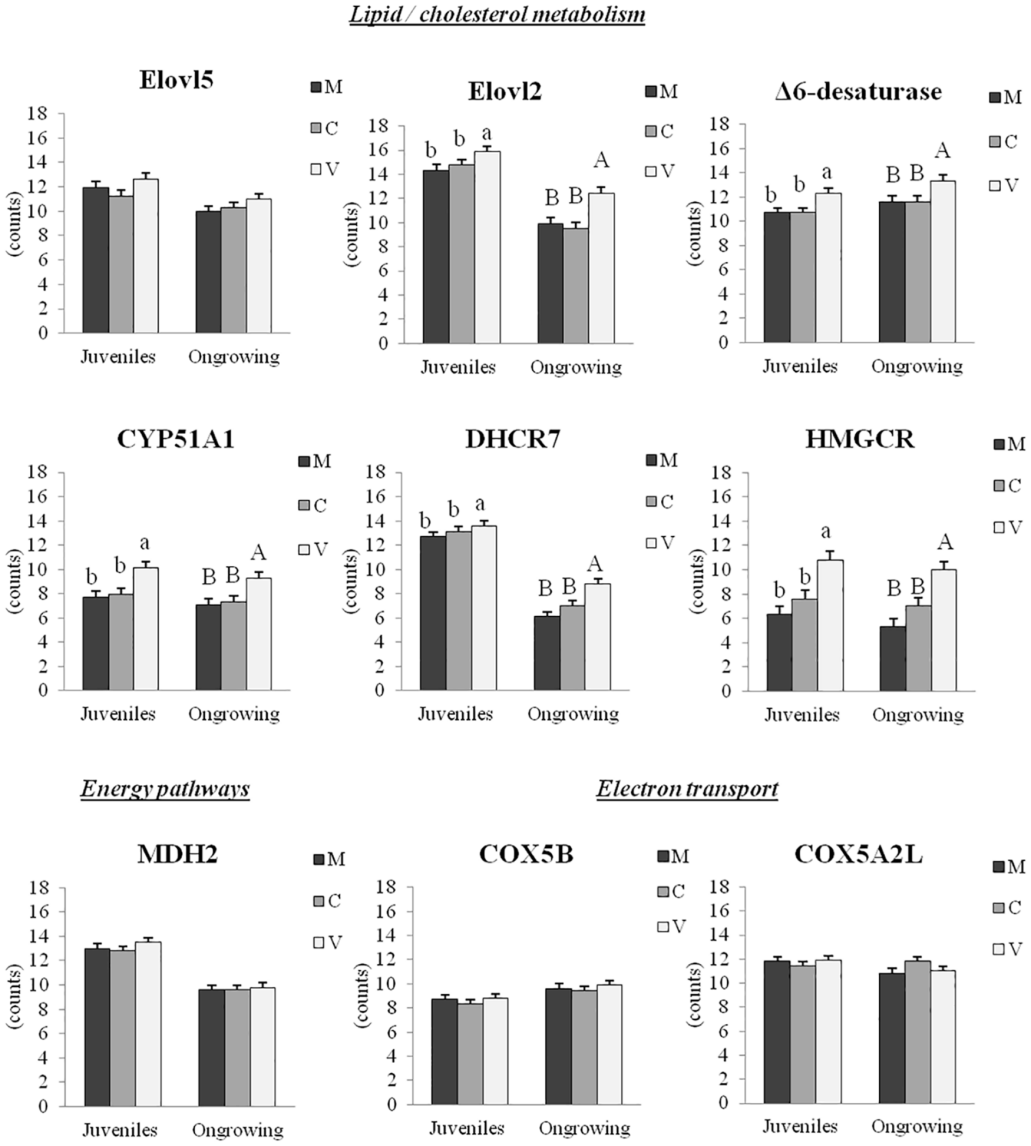
Hepatic gene expression in juveniles and ongrowing fish (RT-qPCR). Data are mean ± S.D. (n = 6 individuals/treatment). Differences between diets were analyzed by one-way ANOVA, followed by Tukey’s test. Values that do not share a common letter are significantly different (*p<0*.*05*), with lower and upper case letters indicating differences between dietary groups within juveniles and ongrowing fish, respectively.

The intestinal and hepatic expression of genes tested by RT-qPCR is shown in Figs 5 and 6, respectively.

#### Intestinal gene expression

The expression of selected genes was measured by RT-qPCR in order to validate microarray results in juveniles. A good match between RT-qPCR and microarray results was found for most of the genes tested (Cathepsin-S, *CTSS;* 6-Phosphofructo-2-Kinase/Fructose-2,6-Biphosphatase 3, *PFKFB3;* Fatty Acid Amide Hydrolase, *FAAH;* Fatty Acid Elongase 5, *Elovl5*).

For genes involved in protein metabolism, higher levels of expression of *CTSZ* were found in ongrowing fish fed the plant-based diets (*p-value* <0.05) compared to those fed the M-diet, while there was no significant difference between dietary groups in juveniles. In terms of carbohydrate metabolism, *PFKFB3* was down-regulated in juveniles with the inclusion of plant ingredients in the diet (M≥C≥V), while up-regulation was observed in C- and V-fed ongrowing fish. For immune response, *CTSS* was down-regulated in fish fed the V-diet compared to those fed the C-diet at the end of the first period of the feeding trial (V<C). Different diet-induced effects were observed in ongrowing fish, with the *CTSS* expression level enhanced in both C- and V-fed fish compared to those fed the M-diet (V = C>M). With regards to fatty acid catabolism in juveniles, we observed significantly lower expression of *FAAH* in the C-fed group compared to the M-fed group and intermediate levels for the V-fed fish. Enhanced expression of *FAAH* was observed in ongrowing fish fed the plant-based diets, compared to those fed the M-diet. No statistically significant differences were observed between groups in the expression levels of *Elovl5*, for juveniles and ongrowing fish. For *Elovl2*, we observed the highest levels of expression in V-fed fish, while the lowest levels were observed for the C-fed group. Up-regulation of *Elovl2* was found in both C- and V-fed groups of ongrowing fish compared to fish fed the M-diet. Up-regulation of *Δ6-desaturase* was observed in V-fed juveniles and in both C-and V-fed ongrowing fish compared to fish fed the M-diet.

#### Hepatic gene expression

The RT-qPCR performed on the livers of juveniles confirmed the microarray results for genes involved in fatty acid bioconversion (*Elovl2*) and cholesterol biosynthesis (lanosterol 14-alpha demethylase, *CYP51A1*; 7-dehydrocholesterol reductase, *DHCR7*). Among the genes involved in fatty acid bioconversion, *Elovl2* and *Δ6-desaturase* were found to be up-regulated in V-fed juveniles and ongrowing fish when compared to the other experimental groups. No statistically significant diet-induced effect was observed in the expression levels of *Elovl5* for both juvenile and ongrowing fish. Up-regulation of *CYP51A1*, *DHCR7* and *HMGCR* (3-hydroxy-3-methyl-glutaryl-CoA reductase) was found in juveniles fed the V-diet compared to the C- and M-fed fish, and the same expression pattern was observed in ongrowing fish (V>C = M).

In contrast to the results observed with the microarray approach, no statistically significant differences were observed for genes involved in energy pathways (Malate Dehydrogenase 2-NAD, *MDH2*) and electron transport (Cytochrome C Oxidase Subunit Vb, *COX5B*; Cytochrome C Oxidase Subunit VIIa Polypeptide 2 Like, *COX7A2L*) in juveniles or in ongrowing fish.

## Discussion

In the context of sustainable aquaculture, replacing FM and FO with less expensive alternatives and more readily available products such as vegetable sources in farmed fish feeds is becoming common practice [4]. Such dietary replacement is known to affect the metabolic response of fish at different levels, including gene expression. However, the long-term effects of plant-based diets still remain poorly documented.

The primary objective of this study was to establish the long-term effects (7 and 13 months) of feeding rainbow trout a diet that substituted FM and FO with increased levels of plant ingredients, and to document the persistence of these changes over time. To assess these effects at the molecular level, we used a microarray approach combined with RT-qPCR analysis on selected genes.

The relatively low number of genes found to be differentially expressed in intestine and liver of juveniles in response to our experimental diets, in addition to the relatively low overall fold changes (FC) obtained, suggested that diet-induced modifications were minimal. In the following discussion, we focus on the main processes highlighted by the enrichment analysis, particularly with respect to genes involved in metabolism-related biological processes.

### Plant-based diets: Effects on growth and survival

Fish survival was affected by the V-diet only during the first twelve weeks after the first feeding. This result can reflect the fact that the early developmental stages for fish are critical, as they undergo important morphological and physiological changes during this time [34]. At the end of this first rearing period (7-month feeding at 7°C), no significant difference in body weight was observed between groups and these results are in accordance with a previous study which demonstrated the remarkable ability of rainbow trout to survive and grow on a totally plant-based diet, completely devoid of marine ingredients [35]. Nevertheless, at the end of the following six months of feeding at 17°C, we observed differences in growth, with fish fed the V-diet displaying the lowest body weight. Low rearing water temperature is known to depress growth in salmonids, whereas at elevated water temperatures growth performance tends to be enhanced [36,37]. In the present study, the absence of differences at the end of the 7°C-feeding period is most likely linked to the low water temperature. Indeed, when M- and C-fed fish were reared at 17°C and could reach their optimum growth performance, a negative effect of the totally plant-based diet became visible. This result supports findings from previous studies which showed lower growth performance in fish fed diets containing different levels of plant ingredients, mainly linked to reduction in feed intake and/or feed efficiency [5,6,9,38,39]. In our study, lower values for feed efficiency were observed in ongrowing fish fed the V-diet, compared to the M- and C-fed fish. Although not statistically significant, this decrease in FE could explain the lower body weights of V-fed fish observed at the end of the second rearing period.

### Gene expression changes in the intestine

The intestine plays a key role in the digestion and absorption of nutrients and is very sensitive to dietary changes as shown by the modifications induced in intermediary metabolism, apoptosis and immune function in response to the inclusion of plant ingredients in aquafeeds [13,16]. Our transcriptomic analysis of the intestines of juveniles revealed a differential regulation of a certain number of cathepsins involved in protein catabolism (*i*.*e*. *CTSZ*, *CTSH*) and apoptotic processes/immunology (*i*.*e*. *CTSS*). Cathepsins are lysosomal cysteine proteases, which have important metabolic roles in the maintenance of cellular homeostasis in organisms [40,41,42]. In a previous study where Atlantic salmon were fed a diet where FM was replaced by plant ingredients, the authors found up-regulation of *CTSZ* and other cathepsins [13]. These results were associated with a high protein turnover because, in addition to the up-regulation of cathepsins involved in protein degradation, the authors also found a simultaneous increase in the expression of genes involved in protein synthesis [13]. These findings are in contrast to what we observed in juveniles fed the V-diet, but are in complete agreement with the enhanced expression of *CTSZ* we found in ongrowing rainbow trout fed the C- and V-diets, compared to M-fed group. This supports the hypothesis of a high turnover in the intestines of fish fed plant-based diets. Cathepsins contribute also to the presentation of endosomal antigens [43]. Among these, cathepsin-S (*CTSS*), a lysosomal cysteine endopeptidase belonging to the papaine family, regulates immunity, antigen presentation and processing in fish [44,45]. In the present study, *CTSS* was down-regulated in the intestine of V-fed juveniles, compared to the M-fed group, while an opposite effect was observed in ongrowing fish suggesting that plant ingredients affect the immune response in rainbow trout, as previously demonstrated in Atlantic salmon and gilthead sea bream [46,47].

The pathway for fatty acid catabolism was also highly affected by dietary replacement as shown by changes in *FAAH* expression. *FAAH* is a membrane-associated protein that is localized in internal membranes, such as the endoplasmic reticulum, in which it is active. In a study on mammals, *FAAH* was shown to be involved in the regulation of intestinal motility, playing a role in the physiological balance of the intestine [48]. In our study, the up-regulation of *FAAH* observed in ongrowing fish fed the C- and V-diets suggest an increase in intestinal motility when trout are fed plant-based diets over a long term period at a relatively high water temperature. These results indicated an impairment of the intestinal physiological balance and are in accordance with the alterations in nutrient absorption and digestion previously observed in salmonids fed plant-based diets [10,49]. However, further investigations are needed to improve our understanding of the biological and physiological roles of *FAAH* in the intestine of fish.

The introduction of plant ingredients in the diet also affects the metabolism of carbohydrates and in this study we observed an overall down-regulation of several genes encoding enzymes related to sugar digestion, such as fucosidase isoforms and mannosidase. These results are in accordance with the down-regulation of genes involved in sugar degradation observed in Atlantic salmon in response to dietary inclusion of plant ingredients [10]. Previous studies in fish have consistently demonstrated an effect of dietary plant ingredients on the hepatic expression of genes involved in glycolysis, the major route of glucose catabolism [6,50]. *PFKFB3* is a powerful activator of 6-phosphofructo-1-kinase, the rate-limiting enzyme of glycolysis [51]. In our study, the down-regulation of *PFKFB3* in C- and V-fed groups may have been linked to the lower levels of dietary starch in the C- and V-diets (11.5 and 8% DM respectively), compared to that of the M-diet (20.5% DM). Interestingly, we observed a different expression profile for this gene in ongrowing fish, with enhanced expression of *PFKFB3* in C- and V-fed fish. These results may suggest either an adaptation of rainbow trout to plant-based diets during the feeding trial, or a temperature effect, reflecting a greater capacity to utilize starch at higher water temperatures as previously shown in this species [52] and also in sea bream [53].

### Gene expression changes in the liver

The liver is arguably the most important metabolically active tissue that responds to circulating dietary nutrients absorbed through the intestine. Concerning sterol metabolism, the up-regulation of *CYP51A1* and *DHCR7*, in fish fed the V-diet is in agreement with previous studies which showed higher expression levels of genes involved in cholesterol biosynthesis in Atlantic salmon [8] and rainbow trout [6] fed plant-based diets. In addition, the expression of *HMGCR*, a rate-limiting step in sterol biosynthesis, was significantly increased with the V-diet in juveniles and ongrowing trout, confirming our previous findings [29]. The absence of dietary cholesterol input in fish fed the V-diet might explain the up-regulation observed in the cholesterol biosynthetic pathway. Indeed, cholesterol is present in FM and FO, while plant ingredients, such as vegetable oils, are rich in phytosterols [49,54], which can interfere with cholesterol metabolism. The significant hypocholesterolemic effect observed in the present study is in line with the results of previous studies with different dietary replacement levels of FM [55,56] or FO [57] in several fish species. Together, the molecular and physiological results of the present study suggest that rainbow trout copes with the absence of dietary cholesterol supply by increasing the expression of genes involved in the cholesterol biosynthesis irrespective of the developmental stage or rearing conditions.

The introduction of plant ingredients in the diets for juveniles enhanced expression of genes involved in energy pathways such as oxidative phosphorylation (*ATP5B* and *ATP5C1*) and a key mitochondrial component of the Krebs cycle (*MDH2*). These results are in contrast to those reported in the livers of trout fed vegetable oils [14]. However, for *MDH2* RT-qPCR validation failed to confirm this expression pattern. Similarly, the differences in the expression of two genes involved in electron transport (*COX5B* and *COX7A2L*) found in the microarray analysis (*p* = 0.038 and *p* = 0.027, respectively), were not confirmed by RT-qPCR in either juveniles or ongrowing fish. For all three genes, the FC detected by microarray analysis was relatively small (FC <2). The FC is known to be one of the factors contributing to the variation in results obtained by microarray *versus* qPCR. Generally speaking, lower correlations were reported for genes exhibiting FC lower than 2. For example, Morey et al. [58] reported a 1.4 FC as the cutoff below which microarray and qPCR data begin to lose correlation. The lack of differences in gene expression between groups we observed by RT-qPCR analysis may therefore be related to the small FC we recorded. Moreover, a total match between the microarray and the RT-qPCR results should not be expected due to limitations associated with RT-qPCR primers, which do not always exactly match the probe on the array, as previously observed in a study on Atlantic salmon liver [59]. Due to the genome duplication (4G) that occurred in salmonids [60], transcriptomic and gene expression studies are often more challenging than in other species due to the presence of duplicated and highly similar genes whose transcripts might be differentially regulated. The transcriptional effects observed in response to the dietary introduction of plant ingredients are therefore sometimes difficult to confirm when they are weak.

### Gene expression changes in both intestine and liver

Liver and intestine are known to be important actors in FA bioconversion in fish. Previous studies investigating the gene expression response of fish after dietary replacement of FO by vegetable oils have shown that, irrespective of the fish species and the oil used, LC-PUFA synthesis pathway is stimulated in these tissues [8,16,30,59,61]. In accordance with these results we observed in our study an up-regulation of polyunsaturated fatty acid elongase-2 (*Elovl2*) in the intestines and livers of juveniles and ongrowing fish fed the V-diet. In order to corroborate our results, we also investigated the expression of two other genes (*Δ6-desaturase* and *Elovl5*) that have key roles in fatty acid bioconversion [62,63]. The up-regulation of *Δ6-desaturase* we found in intestine and liver of V-fed fish support the results of several studies in different fish species [64]. Moreover, they are consistent with the increased activity of the LC-PUFA pathway observed in rainbow trout hepatocytes and enterocytes in response to vegetable oils-based diets [64,65]. In addition, the increase in the quantities of EPA+DHA (g fish^-1^) we found from juveniles to ongrowing V-fed fish provides further evidence that rainbow trout have the capacity to synthesize LC-PUFA from dietary precursors. Indeed, with the V-diet, EPA and DHA intake was zero right from the first feeding and therefore all the EPA and DHA recovered in body lipids come from neo-synthesis.

On the other hand, intestinal and hepatic expression of *Elovl5* did not significantly change in response to diet for juveniles or for ongrowing fish. These results are somewhat surprising, given the importance of this enzyme in LC-PUFA biosynthesis [66]. Still, it is known that both *Elovl5* and *Elovl2* have roles in the elongation of C18 into longer C-chains and that *Elovl5* does not have the capacity to elongate beyond C22 [67]. Given the crucial importance of DHA in fish, it can be hypothesized that the higher expression of *Elovl2* in fish fed the V-diet is linked to a preference given to the biosynthesis of DHA, rather than to EPA. Moreover, it has been shown that EPA can also represent a substrate for DHA production [68]. This increased biosynthesis of DHA is confirmed by the higher percentages of DHA (2% of total FA) we found in whole body juveniles and ongrowing fish, compared to EPA (1% of total FA). However, as previously demonstrated [69], the enhancement of LC-PUFA biosynthesis was not enough to compensate for the lack of provision of dietary n-3 LC-PUFAs. This was reflected in the whole body FA profile, which mirrored the composition of the different diets. Consequently, lower proportions of EPA and DHA were found in body lipids of juveniles and ongrowing fish fed the V-diet compared to those fed the other two diets.

## Conclusions

This study shows a slight effect of a totally plant-based diet on metabolism of rainbow trout fed such a diet from first feeding onwards. This result is supported by the relatively low proportion of differentially expressed metabolism-related genes in the intestine and liver transcriptomes of juveniles. The present work largely confirms the results of previous studies performed over a shorter feeding period, especially with regards to changes in the expression of genes involved in the bioconversion of cholesterol and fatty acids. Our study shows that dietary-induced molecular and biochemical changes in lipid metabolism were maintained in the long term. In contrast, for several genes involved in protein catabolism, immunity and fatty acid catabolism we observed a differential regulation that could be related to the different developmental stages and/or to the differences in rearing temperature. We also characterize new molecular actors affected by the nutritional stress induced in the fish intestine by introducing plant ingredients in diets for rainbow trout, especially for genes involved in intestinal motility. Our results provide a framework for the development of new plant-based feeds to further reduce the reliance of aquaculture on marine fishery resources.

## Supporting information

S1 Table Impact of dietary treatments on the intestinal transcriptome of juveniles. Genes tested by RT-q PCR are in bold.
(DOCX)

S2 Table Impact of dietary treatments on the hepatic transcriptome of juveniles. Genes tested by RT-q PCR are in bold.
(DOCX)

## References

[1] FAO (2016) The State of World Fisheries and Aquaculture 2016. Contributing to food security and nutrition for all Rome 200 pp.

[2] Naylor RL, Hardy RW, Bureau DP, Chiu A, Elliott M, Farrell AP, et al (2009) Feeding aquaculture in an era of finite resources. Proceedings of the National Academy of Sciences 106: 15103–15110. 10.1073/pnas.0905235106 PMC2741212 19805247

[3] Jobling M (2015) Fish nutrition research: past, present and future. Aquaculture International 24: 767–786.

[4] Gatlin DM, Barrows FT, Brown P, Dabrowski K, Gaylord TG, Hardy RW, et al (2007) Expanding the utilization of sustainable plant products in aquafeeds: a review. Aquaculture Research 38: 551–579.

[5] Torstensen B, Espe M, Sanden M, Stubhaug I, Waagbø R, Hemre GI, et al (2008) Novel production of Atlantic salmon (*Salmo salar*) protein based on combined replacement of fish meal and fish oil with plant meal and vegetable oil blends. Aquaculture 285: 193–200.

[6] Panserat S, Hortopan G, Plagnes-Juan E, Kolditz C, Lansard M, Skiba-Cassy S, et al (2009) Differential gene expression after total replacement of dietary fish meal and fish oil by plant products in rainbow trout (*Oncorhynchus mykiss*) liver. Aquaculture 294: 123–131.

[7] Turchini GM, Torstensen BE, Ng WK (2009) Fish oil replacement in finfish nutrition. Reviews in Aquaculture 1: 10–57.

[8] Leaver MJ, Villeneuve LA, Obach A, Jensen L, Bron JE, Tocher DR, et al (2008) Functional genomics reveals increases in cholesterol biosynthetic genes and highly unsaturated fatty acid biosynthesis after dietary substitution of fish oil with vegetable oils in Atlantic salmon (*Salmo salar*). BMC Genomics 9: 299 doi: 10.1186/1471-2164-9-299 18577222 10.1186/1471-2164-9-299 PMC2459193

[9] Benedito-Palos L, Navarro JC, Sitjà-Bobadilla A, Bell JG, Kaushik S, Pérez-Sanchez J (2008) High levels of vegetable oils in plant protein-rich diets fed to gilthead sea bream (*Sparus aurata* L.): growth performance, muscle fatty acid profiles and histological alterations of target tissues. The British Journal of Nutrition 100: 992–1003. doi: 10.1017/S0007114508966071 18377678 10.1017/S0007114508966071

[10] De Santis C, Bartie KL, Olsen RE, Taggart JB, Tocher DR (2015) Nutrigenomic profiling of transcriptional processes affected in liver and distal intestine in response to a soybean meal-induced nutritional stress in Atlantic salmon (*Salmo salar*). Comparative Biochemistry and Physiology Part D: Genomics and Proteomics 15: 1–11. 10.1016/j.cbd.2015.04.001 25916579

[11] Gu M, Kortner TM, Penn M, Hansen AK, Krogdahl Å (2014) Effects of dietary plant meal and soya-saponin supplementation on intestinal and hepatic lipid droplet accumulation and lipoprotein and sterol metabolism in Atlantic salmon (*Salmo salar* L.). The British Journal of Nutrition 111: 432–444. doi: 10.1017/S0007114513002717 24507758 10.1017/S0007114513002717

[12] Taggart JB, Bron JE, Martin SA, Seear PJ, Høyheim B, Talbot R, et al (2008) A description of the origins, design and performance of the TRAITS–SGP Atlantic salmon *Salmo salar* L. cDNA microarray. Journal of Fish Biology 72: 2071–2094. doi: 10.1111/j.1095-8649.2008.01876.x 19125201 10.1111/j.1095-8649.2008.01876.x PMC2610384

[13] Tacchi L, Secombes CJ, Bickerdike R, Adler MA, Venegas C, Takle H, et al (2012) Transcriptomic and physiological responses to fishmeal substitution with plant proteins in formulated feed in farmed Atlantic salmon (*Salmo salar*). BMC Genomics 13: 363 doi: 10.1186/1471-2164-13-363 22853566 10.1186/1471-2164-13-363 PMC3526460

[14] Panserat S, Kolditz C, Richard N, Plagnes-Juan E, Piumi F, Esquerré D, et al (2008) Hepatic gene expression profiles in juvenile rainbow trout (*Oncorhynchus mykiss*) fed fishmeal or fish oil-free diets. The British Journal of Nutrition 100: 953–967. doi: 10.1017/S0007114508981411 18439330 10.1017/S0007114508981411

[15] Geay F, Ferraresso S, Zambonino-Infante JL, Bargelloni L, Quentel C, Vandeputte M, et al (2011) Effects of the total replacement of fish-based diet with plant-based diet on the hepatic transcriptome of two European sea bass (*Dicentrarchus labrax*) half-sibfamilies showing different growth rates with the plant-based diet. BMC Genomics 12: 522 doi: 10.1186/1471-2164-12-522 22017880 10.1186/1471-2164-12-522 PMC3377934

[16] Morais S, Silva T, Cordeiro O, Rodrigues P, Guy DR, Bron JE, et al (2012) Effects of genotype and dietary fish oil replacement with vegetable oil on the intestinal transcriptome and proteome of Atlantic salmon (*Salmo salar*). BMC Genomics 13: 448 doi: 10.1186/1471-2164-13-448 22943471 10.1186/1471-2164-13-448 PMC3460786

[17] Frøystad M, Lilleeng E, Bakke-McKellep A, Vekterud K, Hemre GI, Krogdahl Å (2008) Gene expression in distal intestine of Atlantic salmon (*Salmo salar* L.) fed genetically modified soybean meal. Aquaculture Nutrition 14: 204–214.

[18] Morais S, Edvardsen RB, Tocher DR, Bell JG (2012) Transcriptomic analyses of intestinal gene expression of juvenile Atlantic cod (*Gadus morhua*) fed diets with Camelina oil as replacement for fish oil. Comparative Biochemistry and Physiology Part B: Biochemistry and Molecular Biology 161: 283–293. 10.1016/j.cbpb.2011.12.004 22198123

[19] Calduch-Giner JA, Sitjà-Bobadilla A, Davey GC, Cairns MT, Kaushik S, Pérez-Sánchez J (2012) Dietary vegetable oils do not alter the intestine transcriptome of gilthead sea bream (*Sparus aurata*), but modulate the transcriptomic response to infection with *Enteromyxum leei*. BMC Genomics 13: 470 doi: 10.1186/1471-2164-13-470 22967181 10.1186/1471-2164-13-470 PMC3444936

[20] Murray HM, Lall SP, Rajaselvam R, Boutilier LA, Blanchard B, Flight RM, et al (2010) A nutrigenomic analysis of intestinal response to partial soybean meal replacement in diets for juvenile Atlantic halibut, *Hippoglossus hippoglossus*, L. Aquaculture 298: 282–293.

[21] Kendall AW, Ahlstrom EH, Moser HG (1984) Early life history stages of fishes and their characters In: Moser HG, Richards WJ, Cohen DM, Fahay MP, Kendall AW and Richardson SL, editors. Ontogeny and Systematics of Fishes. American Society of Ichthyologists and Herpetologists Special Publication 1. Lawrence (USA): Allen Press; 1984. p. 11–24.

[22] Woynarovich A, Hoitsy G, Moth-Poulsen T (2011) Small-scale rainbow trout farming: Food and Agriculture Organization of the United Nations. Fisheries and Aquaculture Technical Paper No. 561. Rome. FAO.2011.

[23] Lazzarotto V, Corraze G, Leprevost A, Quillet E, Dupont-Nivet M, Médale F (2015) Three-Year Breeding Cycle of Rainbow Trout (*Oncorhynchus mykiss*) Fed a Plant-Based Diet, Totally Free of Marine Resources: Consequences for Reproduction, Fatty Acid Composition and Progeny Survival. PloS One 10: e0117609 doi: 10.1371/journal.pone.0117609 25658483 10.1371/journal.pone.0117609 PMC4320095

[24] AOAC. Official Methods of Analysis of the AOAC international 17th edition Washington, DC: Association of Official Analytical Chemists; 2000.

[25] McCleary BV, Solah V, Gibson TS (1994) Quantitative measurement of total starch in cereal flours and products. Journal of Cereal Science, 20, 51–58.

[26] Stadtman T (1957) Preparation and assay of cholesterol and ergosterol In: Colowich SP, Kaplan MO, editors. Methods in enzymology 3 New York: Academic Press; 1957. p.392–394.

[27] Folch J, Lees M, Sloane-Stanley G (1957) A simple method for the isolation and purification of total lipids from animal tissues. The Journal of Biological Chemistry 226: 497–509. 13428781

[28] Shantha NC, Ackman RG (1990) Nervonic acid versus tricosanoic acid as internal standards in quantitative gas chromatographic analyses of fish oil longer-chain n—3 polyunsaturated fatty acid methyl esters. Journal of Chromatography: Biomedical Applications 533: 1–10. 2150519 10.1016/s0378-4347(00)82182-9

[29] Lazzarotto V, Corraze G, Larroquet L, Mazurais D, Médale F (2016) Does broodstock nutritional history affect the response of progeny to different first-feeding diets? A whole-body transcriptomic study of rainbow trout alevins. The British Journal of Nutrition 115: 2079–2092. doi: 10.1017/S0007114516001252 27112276 10.1017/S0007114516001252

[30] Morais S, Pratoomyot J, Torstensen BE, Taggart JB, Guy DR, Bell JG, et al (2011) Diet × genotype interactions in hepatic cholesterol and lipoprotein metabolism in Atlantic salmon (*Salmo salar*) in response to replacement of dietary fish oil with vegetable oil. The British Journal of Nutrition 106: 1457–1469. doi: 10.1017/S0007114511001954 21736795 10.1017/S0007114511001954

[31] Olsvik PA, Lie KK, Jordal A-EO, Nilsen TO, Hordvik I (2005) Evaluation of potential reference genes in real-time RT-PCR studies of Atlantic salmon. BMC Molecular Biology 6: 21 doi: 10.1186/1471-2199-6-21 16293192 10.1186/1471-2199-6-21 PMC1314898

[32] Matz MV, Wright RM, Scott JG (2013) No control genes required: Bayesian analysis of qRT-PCR data. PloS One 8: e71448 doi: 10.1371/journal.pone.0071448 23977043 10.1371/journal.pone.0071448 PMC3747227

[33] Hosack DA, Dennis G Jr, Sherman BT, Lane HC, Lempicki RA (2003) Identifying biological themes within lists of genes with EASE. Genome Biology 4: R70 doi: 10.1186/gb-2003-4-10-r70 14519205 10.1186/gb-2003-4-10-r70 PMC328459

[34] Gatesoupe FJ, Zambonino Infante JL, Cahu C, Bergot P (2001) Ontogeny, development and digestive physiology of fish larvae In: Guillaume J, Kaushik S, Bergot P, Métailler R, editors. Nutrition and feeding of fish and crustaceans. Chichester, UK: Springer, Praxis Publishing; 2001. p.197–212.

[35] Le Boucher R, Quillet E, Vandeputte M, Lecalvez JM, Goardon L, Chatain B, et al (2011) Plant-based diet in rainbow trout (*Oncorhynchus mykiss* Walbaum): Are there genotype-diet interactions for main production traits when fish are fed marine *vs* plant-based diets from the first meal? Aquaculture 321: 41–48.

[36] Li M, Leatherland J (2008) Temperature and ration effects on components of the IGF system and growth performance of rainbow trout (*Oncorhynchus mykiss*) during the transition from late stage embryos to early stage juveniles. General and Comparative Endocrinology 155: 668–679. doi: 10.1016/j.ygcen.2007.08.017 17937932 10.1016/j.ygcen.2007.08.017

[37] Baum D, Laughton R, Armstrong J, Metcalfe N (2005) The effect of temperature on growth and early maturation in a wild population of Atlantic salmon parr. Journal of Fish Biology 67: 1370–1380.

[38] Médale F, Kaushik S (2009) Protein sources in feed for farmed fish. Cahiers Agricultures 18: 103–111.

[39] Corraze G, Kaushik S (2009) Lipid nutrition and fish oil replacement by vegetable oils in pisciculture. Cahiers Agricultures 18: 112–118.

[40] Brix K, Dunkhorst A, Mayer K, Jordans S (2008) Cysteine cathepsins: cellular roadmap to different functions. Biochimie 90: 194–207. doi: 10.1016/j.biochi.2007.07.024 17825974 10.1016/j.biochi.2007.07.024

[41] Turk V, Turk B, Turk D (2001) Lysosomal cysteine proteases: facts and opportunities. The EMBO Journal 20: 4629–4633. doi: 10.1093/emboj/20.17.4629 11532926 10.1093/emboj/20.17.4629 PMC125585

[42] Chapman HA, Riese RJ, Shi G-P (1997) Emerging roles for cysteine proteases in human biology. Annual Review of Physiology 59: 63–88. doi: 10.1146/annurev.physiol.59.1.63 9074757 10.1146/annurev.physiol.59.1.63

[43] Hsing LC, Rudensky AY (2005) The lysosomal cysteine proteases in MHC class II antigen presentation. Immunological Reviews 207: 229–241. doi: 10.1111/j.0105-2896.2005.00310.x 16181340 10.1111/j.0105-2896.2005.00310.x

[44] Riese RJ, Mitchell RN, Villadangos JA, Shi G-P, Palmer JT, Karp ER, et al (1998) Cathepsin S activity regulates antigen presentation and immunity. Journal of Clinical Investigation 101: 2351–2363. doi: 10.1172/JCI1158 9616206 10.1172/JCI1158 PMC508824

[45] Zhou J, Li L, Cai Z-H (2012) Identification of putative cathepsin S in mangrove red snapper *Lutjanus argentimaculatus* and its role in antigen presentation. Developmental & Comparative Immunology 37: 28–38. 22210546 10.1016/j.dci.2011.12.011

[46] Lilleeng E, Penn MH, Haugland Ø, Xu C, Bakke AM, Krogdahl Å, et al (2009) Decreased expression of TGF-β, GILT and T-cell markers in the early stages of soybean enteropathy in Atlantic salmon (*Salmo salar* L.). Fish & Shellfish Immunology 27: 65–72. 19427383 10.1016/j.fsi.2009.04.007

[47] Kokou F, Adamidou S, Karacostas I, Sarropoulou E (2016) Sample size matters in dietary gene expression studies—A case study in the gilthead sea bream (*Sparus aurata* L.). Aquaculture Reports 3: 82–87.

[48] Capasso R, Matias I, Lutz B, Borrelli F, Capasso F, Marsicano G, et al (2005) Fatty acid amide hydrolase controls mouse intestinal motility in vivo. Gastroenterology 129: 941–951. doi: 10.1053/j.gastro.2005.06.018 16143133 10.1053/j.gastro.2005.06.018

[49] Krogdahl Å, Penn M, Thorsen J, Refstie S, Bakke AM (2010) Important antinutrients in plant feedstuffs for aquaculture: an update on recent findings regarding responses in salmonids. Aquaculture Research 41: 333–344.

[50] Panserat S, Médale F, Blin C, Breque J, Vachot C, Plagnes-Juan E, et al (2000) Hepatic glucokinase is induced by dietary carbohydrates in rainbow trout, gilthead seabream, and common carp. American Journal of Physiology-Regulatory, Integrative and Comparative Physiology 278: R1164–R1170. doi: 10.1152/ajpregu.2000.278.5.R1164 10801283 10.1152/ajpregu.2000.278.5.R1164

[51] Atsumi T, Nishio T, Niwa H, Takeuchi J, Bando H, Shimizu C, et al (2005) Expression of inducible 6-phosphofructo-2-kinase/fructose-2, 6-bisphosphatase/PFKFB3 isoforms in adipocytes and their potential role in glycolytic regulation. Diabetes 54: 3349–3357. 16306349 10.2337/diabetes.54.12.3349

[52] Brauge C, Corraze G, Médale F (1995) Effect of dietary levels of lipid and carbohydrate on growth performance, body composition, nitrogen excretion and plasma glucose levels in rainbow trout reared at 8 or 18°C. Reproduction Nutrition Development 35: 277–290. 10.1051/rnd:19950304 7612167

[53] Couto A, Enes P, Peres H, Oliva-Teles A (2008) Effect of water temperature and dietary starch on growth and metabolic utilization of diets in gilthead sea bream (*Sparus aurata*) juveniles. Comparative Biochemistry and Physiology Part A: Molecular and Integrative Physiology 151: 45–50. 10.1016/j.cbpa.2008.05.013 18586542

[54] Phillips KM, Ruggio DM, Toivo JI, Swank MA, Simpkins AH (2002) Free and esterified sterol composition of edible oils and fats. Journal of Food Composition and Analysis 15: 123–142.

[55] Sitjà-Bobadilla A, Peña-Llopis S, Gómez-Requeni P, Médale F, Kaushik S, Pérez-Sánchez J, et al (2005) Effect of fish meal replacement by plant protein sources on non-specific defence mechanisms and oxidative stress in gilthead sea bream (*Sparus aurata*). Aquaculture 249: 387–400.

[56] Kaushik S, Cravedi J, Lalles J, Sumpter J, Fauconneau B, Laroche M (1995) Partial or total replacement of fish meal by soybean protein on growth, protein utilization, potential estrogenic or antigenic effects, cholesterolemia and flesh quality in rainbow trout, *Oncorhynchus mykiss*. Aquaculture 133: 257–274.

[57] Richard N, Mourente G, Kaushik S, Corraze G (2006) Replacement of a large portion of fish oil by vegetable oils does not affect lipogenesis, lipid transport and tissue lipid uptake in European seabass (*Dicentrarchus labrax* L.). Aquaculture 261: 1077–1087.

[58] Morey JS, Ryan JC, Van Dolah FM (2006) Microarray validation: factors influencing correlation between oligonucleotide microarrays and real-time PCR. Biological Procedures Online 8: 175–193. doi: 10.1251/bpo126 17242735 10.1251/bpo126 PMC1779618

[59] Morais S, Pratoomyot J, Taggart JB, Bron JE, Guy DR, Bell JG, et al (2011) Genotype-specific responses in Atlantic salmon (*Salmo salar*) subject to dietary fish oil replacement by vegetable oil: a liver transcriptomic analysis. BMC Genomics 12: 255 doi: 10.1186/1471-2164-12-255 21599965 10.1186/1471-2164-12-255 PMC3113789

[60] Allendorf FW, Thorgaard GH (1984) Tetraploidy and the evolution of salmonid fishes In: Turner BJ, editor. Evolutionary genetics of fish. New York: Plenum Press; 1984. p.1–53.

[61] Limtipsuntorn U, Haga Y, Kondo H, Hirono I, Satoh S (2014) Microarray analysis of hepatic gene expression in juvenile Japanese flounder *Paralichthys olivaceus* fed diets supplemented with fish or vegetable oils. Marine Biotechnology 16: 88–102. doi: 10.1007/s10126-013-9535-y 24052493 10.1007/s10126-013-9535-y

[62] Bell M, Dick J, Porter A (2003) Pyloric ceca are significant sites of newly synthesized 22∶6 n−3 in rainbow trout (*Oncorhynchus mykiss*). Lipids 38: 39–44. 12669818 10.1007/s11745-003-1029-5

[63] Fonseca-Madrigal J, Bell JG, Tocher DR (2006) Nutritional and environmental regulation of the synthesis of highly unsaturated fatty acids and of fatty-acid oxidation in Atlantic salmon (*Salmo salar* L.) enterocytes and hepatocytes. Fish Physiology and Biochemistry 32: 317–328.

[64] Torstensen BE, Tocher DR (2011) The effects of fish oil replacement on lipid metabolism of fish In: Turchini GM, Ng W-K, Tocher DR, editors. Fish oil replacement and alternative lipid sources in aquaculture feeds. Boca Raton, FL: CRC Press; 2011. p.405–438.

[65] Fonseca-Madrigal J, Karalazos V, Campbell PJ, Bell JG, Tocher DR (2005) influence of dietary palm oil on growth, tissue fatty acid compositions and fatty acid metabolism in liver and intestine in rainbow trout (*Oncorhynchus mykiss*). Aquaculture Nutrition 11: 241–250.

[66] Morais S, Monroig O, Zheng X, Leaver MJ, Tocher DR (2009) Highly unsaturated fatty acid synthesis in Atlantic salmon: characterization of ELOVL5-and ELOVL2-like elongases. Marine Biotechnology 11: 627–639. doi: 10.1007/s10126-009-9179-0 19184219 10.1007/s10126-009-9179-0

[67] Tocher DR (2003) Metabolism and functions of lipids and fatty acids in teleost fish. Reviews in Fisheries Science 11: 107–184.

[68] Sprecher H, Luthria DL, Mohammed B, Baykousheva SP (1995) Reevaluation of the pathways for the biosynthesis of polyunsaturated fatty acids. Journal of Lipid Research 36: 2471–2477. 8847474

[69] Turchini GM, Francis DS (2009) Fatty acid metabolism (desaturation, elongation and β-oxidation) in rainbow trout fed fish oil-or linseed oil-based diets. The British Journal of Nutrition 102: 69–81. doi: 10.1017/S0007114508137874 19123959 10.1017/S0007114508137874

