# Supplementary material for: Long-term dietary replacement of fishmeal and fish oil in diets for rainbow trout (Oncorhynchus mykiss): Effects on growth, whole body fatty acids and intestinal and hepatic gene expression
Source: PLoS One. 2018 Jan 24;13(1):e0190730. doi: 10.1371/journal.pone.0190730 (PMC5783356; doi:10.1371/journal.pone.0190730)
Supplement: S1 Table — Genes tested by RT-q PCR are in bold. (DOCX) [file pone.0190730.s001.docx]

**Supporting information 1.**

**S1Table. Impact of dietary treatments on the intestinal transcriptome of juveniles**. Genes tested by RT-q PCR are in bold.

|  |  |  | Fold Change (FC) | | |  | ***Significance*** |
| --- | --- | --- | --- | --- | --- | --- | --- |
| **Probe Name** | **Gene Symbol** | **Description** | ***C vs M*** | ***V vs M*** | ***V vs C*** |  | ***p-value*** |
| ***Biological Processes*** |  |  |  |  |  |  |  |
| *Protein catabolism* |  |  |  |  |  |  |  |
| *TC99247* | CTSH | cathepsin H | - 5.1 | - 2.6 | + 2.0 |  | *0.035* |
| *CUST_68_PI425708691* | CTSL2 | cathepsin L2 | - 11. 1 | - 4.0 | + 2.8 |  | *0.041* |
| ***CUST_24029_PI425536763*** | **CTSZ** | **cathepsin Z** | **- 5.7** | **- 2.9** | **+ 2.0** |  | ***0.013*** |
| *TC106655* | DPP7 | dipeptidyl-peptidase 7 | - 2.2 | - 2.1 | + 1.0 |  | *0.036* |
| *CUST_20321_PI425536763* | FOLH1 | folate hydrolase | - 3.9 | - 1.8 | + 2.2 |  | *0.041* |
| *TC110997* | LGMN | legumain | - 9.0 | - 4.5 | + 2.0 |  | *0.041* |
| *CUST_25677_PI425536763* | ENPEP | glutamyl aminopeptidase (aminopeptidase A) | -13.8 | - 1.9 | + 7.2 |  | *0.041* |
| *Carbohydrate metabolism* |  |  |  |  |  |  |  |
| *CUST_21158_PI425536763* | MAN2B1 | mannosidase, alpha, class 2B, member 1 | - 7.1 | - 5.4 | + 1.3 |  | *0.012* |
| *CUST_17398_PI425536763* | FUCA1 | fucosidase, alpha-L- 1, tissue | - 4.4 | - 2.8 | + 1.5 |  | *0.035* |
| *CUST_12758_PI425536763* | FUCA2 | fucosidase, alpha-L- 2, plasma | - 8.3 | - 3.8 | + 2.2 |  | *0.025* |
| *TC114862* | GLB1 | galactosidase, beta 1 | - 11.4 | - 3.5 | + 3.3 |  | *0.016* |
| *TC104967* | NAGA | N-acetyl galactosaminidase, alpha | - 11.2 | - 3.8 | + 3.0 |  | *0.042* |
| *TC108468* | NEU1 | neuraminidase 1 | - 25.8 | - 7.2 | + 3.6 |  | *0.025* |
| **TC123951** | **PFKFB3** | **6-phosphofructo-2-kinase/fructose-2,6-biphosphatase 3** | **- 2.8** | **- 3.0** | **+ 1.1** |  | ***0.042*** |
| *Immune response/ apoptotic process* |  |  |  |  |  |  |  |
| ***CUST_8157_PI425536763*** | **CTSS** | **cathepsin S** | **- 11.0** | **- 3.8** | **+ 2.9** |  | ***0.041*** |
| *CUST_14242_PI425536763* | MPO | myeloperoxidase | -1.5 | - 4.9 | - 3.3 |  | *0.035* |
| *CUST_7188_PI425536763* | BAD | BCL2-associated agonist of cell death | - 2.3 | - 1.3 | + 1.8 |  | *0.045* |
| *TC104795* | MAP3K7 | mitogen-activated protein kinase kinase kinase 7 | - 1.1 | - 1.5 | + 1.6 |  | *0.049* |
| *Fatty acid catabolism* |  |  |  |  |  |  |  |
| ***TC94736*** | CPT1A | carnitine palmitoyltransferase 1A (liver) | + 1.4 | + 1.0 | - 1.4 |  | *0.045* |
| ***TC121737*** | **FAAH** | **fatty acid amide hydrolase** | **- 18.6** | **- 3.0** | **+ 6.2** |  | ***0.041*** |
| *Transcription and activation* |  |  |  |  |  |  |  |
| *TC125816* | PQBP1 | polyglutamine binding protein 1 | + 1.3 | - 1.1 | - 1.4 |  | *0.042* |
| *Miscellaneous* |  |  |  |  |  |  |  |
| *CUST_17716_PI425536763* | MXD4 | Max dimerization protein 4 | - 1.7 | - 1.6 | + 1.1 |  | *0.025* |
| *CUST_1243_PI425536763* | POLR2F | polymerase (RNA) II (DNA directed) polypeptide F | + 1.4 | - 1.1 | - 1.5 |  | *0.039* |
| *TC118891* | PRPSAP2 | phosphoribosyl pyrophosphate synthetase-associated protein 2 | + 1.4 | - 1.0 | - 1.4 |  | *0.025* |
| *CUST_9923_PI425536763* | TTC4 | tetratricopeptide repeat domain 4 | + 1.8 | + 1.5 | - 1.2 |  | *0.039* |
| *CUST_6882_PI425536763* | RENBP | renin binding protein | - 4.4 | - 2.5 | + 1.8 |  | *0.036* |
| *TC95545* | ASAH1 | N-acylsphingosine amidohydrolase 1 | - 2.6 | - 1.9 | + 1.4 |  | *0.041* |
| *CUST_15445_PI425536763* | ASH2L | ash2 (absent, small, or homeotic)-like | - 2.2 | - 1.7 | + 1.3 |  | *0.049* |
|  |  |  |  |  |  |  |  |
| ***Molecular function*** |  |  |  |  |  |  |  |
| *CUST_17473_PI425536763* | ABCC12 | ATP-binding cassette, sub-family C (CFTR/MRP), member 12 | - 2.1 | - 2.1 | + 1.0 |  | *0.049* |
| *TC108892* | ABCC4 | ATP-binding cassette, sub-family C (CFTR/MRP), member 4 | - 5.0 | - 2.0 | + 2.5 |  | *0.039* |
| *TC104435* | ARHGAP12 | Rho GTPase activating protein 12 | - 2.1 | - 1.4 | + 1.5 |  | *0.049* |
| *CUST_6855_PI425536763* | ATP2C1 | ATPase, Ca++ transporting, type 2C, member 1 | - 4.0 | - 3.6 | + 1.1 |  | *0.029* |
| *CUST_18236_PI425536763* | ATP6V0A1 | ATPase, H+ transporting, lysosomal V0 subunit a1 | - 3.2 | - 1.4 | + 2.2 |  | *0.043* |
| *CUST_5593_PI425536763* | ATP6V0C | ATPase, H+ transporting, lysosomal 16kDa, V0 subunit c | - 1.9 | - 1.7 | + 1.1 |  | *0.039* |
| *TC112228* | CDKN1B | cyclin-dependent kinase inhibitor 1B | + 4.8 | + 1.5 | -3.2 |  | *0.046* |
| *TC117615* | CUBN | cubilin (intrinsic factor-cobalamin receptor) | - 45.4 | - 5.0 | + 9.0 |  | *0.042* |
| *CUST_21136_PI425536763* | SLC16A1 | solute carrier family 16, member 1 (monocarboxylic acid transporter 1) | + 1.9 | - 1.4 | - 2.6 |  | *0.049* |
| *CUST_7347_PI425536763* | SNX8 | sorting nexin 8 | - 2.6 | - 1.7 | + 1.5 |  | *0.042* |
| *TC104334* | TTN | titin | - 2.7 | - 1.4 | + 1.9 |  | *0.045* |
|  |  |  |  |  |  |  |  |
| ***Cellular component*** |  |  |  |  |  |  |  |
| *CUST_23323_PI425536763* | ADCY1 | adenylate cyclase 1 | - 1.8 | - 1.6 | + 1.1 |  | *0.042* |
| *TC100687* | PSAP | prosaposin | - 1.8 | - 1.6 | + 1.1 |  | *0.025* |
| *TC128912* | RPS14 | ribosomal protein S14 | - 2.3 | - 2.1 | + 1.1 |  | *0.048* |
| *TC107355* | SNAP23 | synaptosomal-associated protein 23 | - 2.0 | - 1.2 | + 1.7 |  | *0.048* |
| *CUST_5785_PI425536763* | VPS41 | vacuolar protein sorting 41 (yeast) | - 1.9 | - 1.3 | + 1.4 |  | *0.030* |
| *CUST_21742_PI425536763* | ST7 | suppression of tumorigenicity 7 | + 1.3 | - 1.2 | - 1.5 |  | *0.046* |
