# Supplementary material for: Long-term dietary replacement of fishmeal and fish oil in diets for rainbow trout (Oncorhynchus mykiss): Effects on growth, whole body fatty acids and intestinal and hepatic gene expression
Source: PLoS One. 2018 Jan 24;13(1):e0190730. doi: 10.1371/journal.pone.0190730 (PMC5783356; doi:10.1371/journal.pone.0190730)
Supplement: S2 Table — Genes tested by RT-q PCR are in bold. (DOCX) [file pone.0190730.s002.docx]

**Supporting information 2**

**S2 Table. Impact of dietary treatments on the hepatic transcriptome of juveniles.** Genes tested by RT-q PCR are in bold.

|  |  |  | Fold Change (FC) | | |  | ***Significance*** |
| --- | --- | --- | --- | --- | --- | --- | --- |
| **Probe name** | **Gene Symbol** | **Description** | ***C vs M*** | ***V vs M*** | ***V vs C*** |  | ***p-value*** |
| ***Biological processes*** |  |  |  |  |  |  |  |
| *Lipids/Cholesterol Metabolism* | |  |  |  |  |  |  |
| **CUST_14393_PI425536763** | **Elovl2** | **polyunsaturated fatty acid elongase** | **+ 1.4** | **+ 2.6** | **+ 1.8** |  | ***0.049*** |
| **TC130473** | **CYP51A1** | **cytochrome P450, family 51, subfamily A, polypeptide 1** | **+ 2.3** | **+ 4.1** | **+ 1.8** |  | ***0.046*** |
| **TC130143** | **DHCR7** | **7-dehydrocholesterol reductase** | **+ 2.3** | **+ 3.8** | **+ 1.6** |  | ***0.049*** |
| CUST_9914_PI425536763 | TM7SF2 | transmembrane 7 superfamily member 2 | + 2.6 | + 5.7 | + 1.8 |  | *0.030* |
|  |  |  |  |  |  |  |  |
| *Energy pathways* |  |  |  |  |  |  |  |
| CUST_21841_PI425536763 | ATP5B | ATP synthase, H+ transporting mitochondrial F1 complex, beta subunit | - 1.0 | + 1.5 | + 1.5 |  | *0.030* |
| CUST_20841_PI425536763 | ATP5C1 | ATP synthase, H+ transporting, mitochondrial F1 complex, gamma polypeptide 1 | +1.1 | + 1.4 | + 1.3 |  | *0.040* |
| **CUST_11055_PI425536763** | **MDH2** | **malate dehydrogenase 2, NAD (mitochondrial)** | **+1.0** | **+ 1.7** | **+ 1.7** |  | ***0.030*** |
| TC114386 | UQCRC1 | ubiquinol-cytochrome c reductase core protein I | -1.0 | + 1.7 | + 1.7 |  | *0.030* |
|  |  |  |  |  |  |  |  |
| *Electron Transport* |  |  |  |  |  |  |  |
| **TC105004** | **COX5B** | **cytochrome c oxidase subunit Vb** | **-1.2** | **+1.4** | **+ 1.6** |  | ***0.038*** |
| **TC99046** | **COX7A2L** | **cytochrome c oxidase subunit VIIa polypeptide 2 like** | **-1.2** | **+1.5** | **+ 1.9** |  | ***0.027*** |
|  |  |  |  |  |  |  |  |
| ***Molecular function*** |  |  |  |  |  |  |  |
| TC100568 | ACTN3 | actinin alpha 3a | - 1.4 | - 3.6 | - 2.5 |  | *0.027* |
| TC103820 | CSRP1 | cysteine and glycine-rich protein 1a | + 1.0 | - 2.2 | -2,3 |  | *0.046* |
| CUST_8095_PI425536763 | DBI | diazepam binding inhibitor (GABA receptor modulator, acyl-CoA binding protein) | + 1.4 | + 2.3 | + 1.6 |  | *0.030* |
| CUST_10542_PI425536763 | DRG1 | developmentally regulated GTP binding protein 1 | - 1.5 | + 1.5 | + 2.2 |  | *0.027* |
| CUST_21649_PI425536763 | NDUFA9 | NADH dehydrogenase (ubiquinone) 1 alpha subcomplex, 9 | + 1.1 | + 1.5 | + 1.4 |  | *0.046* |
| TC102108 | RAE1 | RNA export 1 homolog (S. pombe) | - 1.2 | + 1.3 | + 1.5 |  | *0.030* |
| *Macromolecule biosynthesis* |  |  |  |  |  |  |  |
| TC100227 | DENR | density-regulated protein | - 1.3 | + 1.3 | + 1.7 |  | *0.049* |
| TC95842 | EIF5 | eukaryotic translation initiation factor 5 | - 1.4 | + 1.4 | + 2.0 |  | *0.033* |
| CUST_7899_PI425536763 | MRPL3 | mitochondrial ribosomal protein L3 | - 1.2 | + 1.3 | + 1.5 |  | *0.030* |
| CUST_3849_PI425536763 | MRPS18B | mitochondrial ribosomal protein S18B | - 1.4 | + 1.2 | + 1.6 |  | *0.030* |
|  |  |  |  |  |  |  |  |
| ***Cellular component*** |  |  |  |  |  |  |  |
| CUST_17684_PI425536763 | SEC23IP | SEC23 interacting protein | - 1.4 | + 1.5 | + 1.4 |  | *0.036* |
| TC112917 | NCOA4 | nuclear receptor coactivator 4 | + 2.4 | - 2.1 | - 5.1 |  | *0.027* |
